# PRDM1-driven SLC30A9 overexpression contributes to the malignant phenotype of cervical cancer cells via promoting mitochondrial hyperfunction

**DOI:** 10.1038/s41419-025-08264-x

**Published:** 2025-12-19

**Authors:** Hui Wang, Shuang Liu, Ping Li, Yu-song Zhang, Yan Zhang, Rong Ma, Juan Wang

**Affiliations:** 1 Department of Abdominal and Pelvic Tumor Treatment, The Second Affiliated Hospital of Soochow University, Suzhou, China; 2 Department of Gynecology, Harbin Medical University Cancer Hospital, Harbin, China; 3 Department of Radiotherapy and Oncology, Affiliated Kunshan Hospital of Jiangsu University, Kunshan, China; 4 Department of Obstetrics and Gynecology, the First Affiliated Hospital of Soochow University, Suzhou, China

**Keywords:** Cervical cancer, Targeted therapies

## Abstract

Mitochondrial hyperfunction is important for cervical cancer progression. Solute carrier family 30 member 9 (SLC30A9) is a member of the solute carrier family 30, specifically a zinc transporter that plays a critical role in mitochondrial zinc homeostasis and maintaining mitochondrial function. We investigated the expression, function, and underlying mechanisms of SLC30A9 in the context of cervical cancer. Single-cell RNA sequencing analysis revealed a marked overexpression of *SLC30A9* within the malignant epithelial cell population of cervical squamous cell carcinoma. This elevated SLC30A9 expression was further corroborated in clinical specimens from local patients and across various established and primary cervical cancer cells. SLC30A9 shRNA or knockout (via CRISPR/Cas9 method) significantly impeded the viability, proliferation, cell cycle progression and migration, and triggered apoptosis in cervical cancer cells. SLC30A9 depletion disrupted mitochondrial function, inhibiting mitochondrial respiration, mitochondrial membrane potential, mitochondrial complex I activity, and ATP production, also caused mitochondrial Zn^2+^ accumulation, reactive oxygen species (ROS) production and oxidative injury. Conversely, overexpression of SLC30A9 in cervical cancer cells demonstrated enhanced mitochondrial complex I activity, increased ATP production, and augmented cellular proliferation and migration. Bioinformatic analysis, coupled with functional validation, identified PRDM1 (PR Domain Containing 1) as a key transcription factor regulating SLC30A9 expression. Silencing or knockout of PRDM1 resulted in a significant reduction in SLC30A9 promoter activity, as well as decreased *SLC30A9* mRNA and protein levels in primary cervical cancer cells. Chromatin immunoprecipitation (ChIP) assays confirmed increased PRDM1 binding to the *SLC30A9* promoter region in cervical cancer tissues. In vivo studies showed that SLC30A9 knockdown led to a remarkable decrease in the growth of xenografts formed by primary cervical cancer cells. These SLC30A9-silenced xenografts exhibited mitochondrial dysfunction, proliferation inhibition and apoptosis induction. These findings collectively suggest that PRDM1-driven SLC30A9 overexpression significantly contributes to the malignant phenotype of cervical cancer, possibly through promoting mitochondrial hyperfunction.

## Introduction

Cervical cancer, a malignancy originating from the cervix uteri, remains a significant global health burden, particularly in developing regions [1, 2]. It is the fourth most common cancer among women worldwide [1, 2]. Persistent infection with high-risk human papillomavirus (HPV) is the primary etiological factor [3]. Current treatment modalities, including surgical resection, radiation therapy, and chemotherapy, are tailored to the stage of disease and patient-specific factors [4–6]. The introduction of HPV vaccination and early-screening programs have significantly reduced incidence rates in many regions [5–8]. However, disparities in access to these interventions persist [5–8]. Prognosis varies widely, with five-year survival rates ranging from 15 to 25% for advanced-stage disease to over 80–90% for early-stage disease [4]. This emphasizes the crucial role of early detection and effective management strategies.

Targeted therapies have shown promise in treating cervical cancer by targeting specific molecules involved in tumor growth [9–12]. Recent advancements have highlighted the role of immune checkpoint inhibitors, such as pembrolizumab and nivolumab, which enhance immune response against HPV-positive cervical cancers by blocking immune checkpoint pathways [9]. In addition, inhibition of angiogenesis through targeted therapies has gained traction. Bevacizumab and other monoclonal antibodies against vascular endothelial growth factor (VEGF) have shown efficacy in combination with chemotherapy, improving progression-free survival in patients with advanced cervical cancer [13, 14]. Research is also exploring the targeting of other molecules like fibroblast growth factor receptor (FGFR) to further improve treatment [15, 16]. While these therapies are promising, identifying patients who will benefit and overcoming drug resistance remains a challenge [9–12]. Therefore, identifying new therapeutic targets is important for advancing cervical cancer treatment [9–12].

Mitochondria are essential organelles in cancer development, responsible for vital functions such as metabolism, survival, growth, and apoptosis regulation [17]. Augmented mitochondrial function/activity plays an important role in the progression of cervical cancer by facilitating enhanced bioenergetic capacity and metabolic flexibility, which are essential for supporting the increased proliferation of cancer cells [18–22]. To meet the increased energy demands of tumor cells, glycolytic activity is significantly elevated [23, 24]. Recent studies indicate that oxidative phosphorylation (OXPHOS) is also enhanced in various cancers [23, 25, 26]. Importantly, elevated OXPHOS can occur alongside high glycolytic activity [23, 25, 26]. In cervical cancer, HPV E7 transfection has been shown to increase OXPHOS levels [27]. Consequently, inhibiting OXPHOS through pharmacological or genetic means has demonstrated significant potential in suppressing the growth of cancer cells, highlighting the therapeutic promise of targeting mitochondrial metabolism in cervical cancer [27].

Zinc exerts profound influence beyond the cytosol, significantly impacting mitochondrial function. Specifically, it modulates key metabolic pathways such as the tricarboxylic acid cycle (TCA) and the electron transport chain [28–30]. Excessive zinc has been shown to disrupt the integrity and activity of electron transport chain components, as well as components involved in glycolysis and the TCA cycle [28–30]. Solute carrier family 30 member 9 (SLC30A9), a mitochondrial zinc transporter, facilitates the efflux of zinc from the mitochondrial matrix to the cytosol [31–33]. Genetic ablation of SLC30A9 resulted in the deleterious accumulation of zinc within mitochondria, leading to pronounced impairments in mitochondrial function [31–33]. Loss of SLC30A9 function led to a significant reduction in mitochondrial ribosomes and OXPHOS proteins, including all six mitochondrial DNA-encoded OXPHOS subunits [34]. This phenotype is comparable to that observed in cells lacking the mitochondrial ribosomal sentinel protein MRPS22 (mitochondrial ribosomal protein S22) [34]. Furthermore, SLC30A9 deficiency resulted in decreased mitochondrial membrane potential, reduced ATP levels, and impaired mitochondrial activity [34]. Given its critical role in mitochondrial function, further investigation into SLC30A9’s involvement in cervical cancer pathogenesis is warranted.

## Materials and methods

### Single-cell RNA sequencing analysis

Single-cell RNA sequencing data from cervical cancer samples (accession number E-MTAB-11948) were obtained from the ArrayExpress database based on the original authors [35]. The single-cell RNA sequencing data were processed using R software, version 4.4.0. The software was downloaded from the Comprehensive R Archive Network (CRAN) website, available at the following link: https://cran.r-project.org/. The dataset was processed using the standard Seurat analysis pipeline. Following quality control, the data were normalized using the NormalizeData function to account for differences in sequencing depth between cells. Highly variable features were identified using the FindVariableFeatures function, selecting the top 2000 genes based on their variance-stabilizing transformation method. The expression of these highly variable genes was then scaled using the ScaleData function, which centers and scales gene expression values. Principal Component Analysis (PCA) was performed on the scaled data to reduce dimensionality. The statistically significant principal components were determined and used for subsequent analysis. Graph-based clustering was performed using the FindNeighbors and FindClusters functions. The FindNeighbors function constructs a k-nearest neighbor graph based on the Euclidean distance in the PCA-reduced space. The FindClusters function then applies the Louvain algorithm to partition the cells into clusters. The resolution parameter for FindClusters was optimized to achieve distinct and biologically meaningful cell populations. Uniform Manifold Approximation and Projection (UMAP) was employed using the RunUMAP function to visualize the cells in a two-dimensional space based on the calculated clusters. Cell clusters were manually annotated based on the expression profiles of known classical marker genes for various cell types present in the cervical tumor microenvironment. The epithelial cell subpopulation was specifically extracted from the complete dataset using the subset function based on the assigned cell type labels. Within this epithelial cell subset, cells positive for the expression of the *SLC30A9* gene were identified. A gene co-expression analysis was conducted within the *SLC30A9*-positive epithelial cell population. Genes with the highest correlation coefficients to *SLC30A9* expression were identified, and the top 100 most positively correlated genes were selected for further analysis. Functional and pathway enrichment analysis of these top 100 co-expressed genes was performed using the Enrichr online tool.

### Reagents and antibodies

Reagents were sourced from Sigma-Aldrich (St. Louis, MO), including puromycin, polybrene, N-acetylcysteine, resveratrol, Paclitaxel (Taxol) fetal bovine serum (FBS) and other cell culture reagents, Matrigel, and the CCK-8 assay kit. Thermo Fisher Scientific (Invitrogen, Carlsbad, CA) provided Terminal deoxynucleotidyl transferase dUTP nick end labeling (TUNEL), 5-ethynyl-2’-deoxyuridine (EdU), 4’,6-diamidino-2-phenylindole (DAPI), JC-1, and all other fluorescent dyes. RhodZin-3, a mitochondrial fluorescent zinc sensor [36], was purchased from Guidechem (Hangzhou, China). The anti-SLC30A9 antibodies were obtained from Sigma-Aldrich (HPA004014, for IHC staining) and Invitrogen (PA5-101969, for Western blot assays). The anti-SLC25A25 polyclonal antibody was also obtained from Invitrogen. The anti-PRDM1 antibody was provided by Cell Signaling Tech. Other antibodies were reported in our previous studies [37, 38]. Genechem (Shanghai, China) synthesized the nucleotide sequences, viral constructs, and PCR primers, unless otherwise mentioned.

### Cells

As described earlier [39, 40], cervical cancer tissues and adjacent paracancerous epithelial tissues were chopped into small pieces and digested with collagenase type I and dispase II. The cells were washed, centrifuged, and incubated in a complete medium with penicillin/streptomycin and DNase (500 U). After filtering and resuspending, primary cervical cancer cells (“pCCa-1” and “pCCa-2” and “pCCa-3”, from three patients) and cervical epithelial cells (HCerEpC) were cultured in high glucose DMEM/F-12 medium with 12% FBS, hydrocortisone, insulin, EGF, and adenine. HeLa cervical cancer cell line and Ect1/E6E7 epithelial cell lines were obtained from the Cell Bank of the Institute of Biological Science at CAS (Shanghai, China). HeLa cells were cultured in DMEM supplemented with 10% FBS, while Ect1/E6E7 epithelial cells were cultured in specialized keratinocyte-serum free medium (K-SFM, Gibco) enriched with Bovine Pituitary Extract (BPE, Gibco), EGF, and supplemental calcium chloride (Gibco). For all cell functional assays, cervical cancer cells or epithelial cells with different genetic modifications/treatments were cultured under low-serum conditions, specifically in basal medium supplemented with 0.5% FBS (fetal bovine serum) and 5.5 mM of glucose. All protocols involving human cells were approved by the Ethics Committee of The Affiliated Kunshan Hospital of Jiangsu University (#KY-2021-096-BR) and adhered to the Helsinki Declaration principles.

### Human tissues

Cervical cancer and matched paracancerous normal epithelial tissues were collected from twenty patients at the Affiliated Hospitals of Soochow University (Suzhou, China) with written informed consent, all provided by Dr. Cao [40]. Relevant patient details were provided earlier [40]. Tissue slides underwent immunohistochemistry (IHC) staining following established protocols [41] or immunofluorescence staining following the reported protocols [42, 43]. The fresh tissue lysates were analyzed using Western blotting and qRT-PCR. All procedures were approved by the Ethics Committee of The Affiliated Kunshan Hospital of Jiangsu University (#KY-2021-096-BR) and adhered to the Helsinki Declaration principles.

### Western blotting

Protein lysates (30–40 µg) from cells and tissues were separated using SDS-PAGE (sodium dodecyl sulfate-polyacrylamide gel electrophoresis) (10–15% gels) and transferred onto PVDF membranes. After blocking, membranes were incubated overnight at 4 °C with primary antibodies, followed by secondary antibodies for 55 min at room temperature. Protein bands were visualized using enhanced chemiluminescence (ECL) under an X-ray machine (LD-14, Suzhou Brooke Flushing Equipment Co., Ltd, Suzhou, China). Results were quantified using ImageJ software (1.50b). Uncropped blot images are shown in Fig. S1.

### Quantitative real-time PCR (qRT-PCR)

RNA was extracted from cells and tissues using TRIzol, reverse transcribed into cDNA using a Takara PCR amplification kit (Beijing, China), and analyzed by qRT-PCR using SYBR Green PCR Master Mixes (Beijing, China) on the ABI-7900 system. *GAPDH* (*glyceraldehyde-3-phosphate dehydrogenase*) was used as the reference gene, and relative gene expression was calculated using the 2^-ΔΔCt^ method. Primers were provided by Genechem.

### Short hairpin RNA (shRNA)

Two different shRNAs against SLC30A9, shSLC30A9-S1 and shSLC30A9-S2, one shRNA against PRDM1 (PR Domain Containing 1), along with a scrambled control shRNA (“shC”, provided by Dr. Cao [40]), were cloned into the GV369 vector from Genechem. Lentiviruses were generated by co-transfecting these constructs with viral packaging plasmids into HEK-293 cells. The resultant lentiviruses were used to infect cervical cancer or epithelial cells. After selection with puromycin, stable cells with SLC30A9 knockdown or PRDM1 knockdown were established.

### CRISPR (clustered regularly interspaced short palindromic repeats)/Cas9 (CRISPR-associated protein 9)-mediated gene knockout

Cervical cancer cells at 60% confluence were cultured in complete medium with polybrene and infected with a Cas9-expressing lentivirus (from Dr. Cao [40, 44]) to create stable cells. These cells were then transduced with constructs encoding sgRNAs (single-guide RNAs) for CRISPR/Cas9-mediated SLC30A9 knockout, each containing unique sgRNA sequences targeting SLC30A9: koSLC30A9-sg1 and koSLC30A9-sg2. After selection with puromycin, the cells were plated at single-cell density in 96-well plates. Successful SLC30A9 knockout was verified by Western blotting analysis. Control cells received a lentiviral vector expressing Cas9 with a nonsense sgRNA (“sgC”), also from Dr. Cao [40, 44]. For PRDM1 KO, the Cas9-expressing pCCa-1 cells were transduced with a CRISPR/Cas9-PRDM1-KO construct encoding an sgRNA sequence against PRDM1. The other steps were the same.

### SLC30A9 overexpression

The hSLC30A9[NM_006345.4]-expressing lentiviral GV369 construct and a vector control (“Vec”) were obtained from Genechem. Lentiviruses were produced by co-transfecting these constructs with virus packaging plasmids into HEK-293 cells. These viral particles infected primary cultured cervical cancer cells, which were then switched to medium with puromycin after 48 h. Stable SLC30A9-overexpressing cells were established after 6 passages, confirmed by qRT-PCR and Western blotting analysis. To generate an shRNA-resistant *SLC30A9* expression construct, a mutant SLC30A9 cDNA containing a sequence resistant to shSLC30A9-S2 was utilized.

### siRNA

pCCa-1 cells were cultured under standard conditions and transfected with verified siRNA at a concentration of 200 nM. The transfection process was performed in two sequential rounds, each lasting 24 h, to ensure efficient gene silencing. The expression levels of the target mRNAs were then quantified by qRT-PCR assays. The SLC25A25 siRNA was obtained from Origene (Beijing, China).

### Flow cytometry

Flow cytometry was utilized to quantitatively assess apoptosis and cell cycle distribution. For apoptosis, cells were stained with the Annexin V-FITC/propidium Iodide (PI) apoptosis detection kit (Thermo-Fisher Scientific, Shanghai, China) according to the manufacturer’s protocol, involving a 15-min incubation at room temperature in the dark. For cell cycle analysis, cells were fixed overnight in cold 70% ethanol at 4 °C, then stained with a solution containing 50 µg/mL PI and 100 µg/mL RNase A for 30 min at 37 °C. All fluorescence measurements were acquired on a BD FACSCanto II flow cytometer, with data acquisition and analysis performed using FlowJo software (v10). The percentages of cells in different cell cycle phases or stages of apoptosis were quantitatively determined from the resulting histograms, with all mean values calculated from five independent biological replicates.

### Other cellular functional studies

Including CCK-8 assay for cell viability, colony formation, “Transwell” assay for cell migration and “Matrigel Transwell” assay for cell invasion, as well as nuclear EdU staining assay for cell proliferation, Caspase-3/Caspase-9 activity assays, Histone-associated DNA ELISA were described in detail in our earlier studies [37, 39, 40]. The quantification for EdU assays involved randomly selecting five fields of view per group, accurately counting both EdU-positive nuclei and total nuclei (DAPI-positive), and calculating the proliferation rate as the ratio of EdU-positive nuclei to the total nuclei count. Mean values were calculated from five biological replicates. The Transwell assays were conducted under standardized conditions, utilizing a precise cell density of 10,000 cells per well and a fixed incubation period of 24 h. At this specific time point, there was no significant change in proliferation/viability/cell number. For quantification, the number of cells that migrated or invaded was determined by counting cells in five random fields per well under a 100× magnification microscope. The average cell count was then calculated from five independent biological replicates.

### Oxygen consumption rate (OCR) and extracellular acidification rate (ECAR)

OCR was quantified using the XF24 Extracellular Flux Analyzer (Agilent Seahorse Bioscience, Billerica, MA) following standard protocols as reported earlier [45]. Cells were subjected to sequential treatment with 1 μM oligomycin, 0.5 μM FCCP (carbonyl cyanide-p-trifluoromethoxyphenylhydrazone), and a combination of 0.5 μM antimycin A and rotenone to determine basal, ATP-linked, maximal, and non-mitochondrial OCR [45]. Measurements were consistently normalized to intracellular protein levels. ECAR was quantified using the XF24 Extracellular Flux Analyzer as well, following standard protocols to assess cellular glycolytic function. In brief, cervical cancer cells were subjected to sequential treatment with 10 mM glucose, 1 μM oligomycin, and 50 mM 2-deoxy-D-glucose (2-DG). This procedure determined the basal glycolysis, glycolytic capacity, and non-glycolytic acidification, respectively. For Seahorse assays, cells were first incubated in glucose-free and no-serum basal medium to establish a baseline metabolic rate. Glucose was only added during the ECAR assay itself. All measurements were consistently normalized to intracellular protein levels. Mean values were calculated from five biological replicates.

### Mitochondrial complex I activity and ATP measurements

The functionality of mitochondrial complex I in cellular and tissue extracts was assessed using a Sigma-Aldrich kit that quantifies the conversion of NADH (nicotinamide adenine dinucleotide) to NAD^+^ (nicotinamide adenine dinucleotide) through spectrophotometric techniques. A decrease in absorbance at 350 nm signified the activity of complex I. Additionally, ATP levels in the cells and tissues were quantified using a colorimetric kit from Sigma, adhering to established protocols. Each analysis utilized 30 μL of lysates, containing 30 μg of total protein. Mean values were calculated from five biological replicates.

### Mitochondrial DNA content

To measure mitochondrial DNA (mtDNA) levels, total DNA was extracted from the cultured cells using the phenol-chloroform method. Quantitative PCR (qPCR) was then performed with primers specific for mitochondrial gene MT-ND1 (NADH dehydrogenase subunit 1) and the nuclear reference gene (GAPDH) to allow normalization of mtDNA content. The reactions were conducted under standard cycling conditions, with melt curve analysis performed to confirm specificity. The relative mtDNA copy number was calculated using the ΔΔCt method comparing the threshold cycle (Ct) values of mitochondrial and nuclear targets. Mean values were calculated from five biological replicates.

### Fluorescence dye assays measuring mitochondrial functions

Following the specified incubation periods, live cervical cancer cells were stained by removing the existing medium and replacing it with a fresh, serum-free basal medium containing the designated fluorescent dye. Following a 30-min incubation at 37 °C, the cells are washed 2 times with warm PBS (37 °C) to remove unbound dye. Cells were then immediately imaged using a fluorescence microscope after the addition of fresh PBS. The images were taken using a ZEISS LSM 900 confocal microscope with 10x (0.45 NA) dry objectives, a resolution of 1024 × 1024 pixels and ZEN 3.4 software for microscope control and image acquisition. Following acquisition, raw fluorescence images (in .czi format) were directly converted as .jpg files without any background subtraction, filtering, or adjustments to brightness and contrast. The original .czi files from the fluorescence microscope were automatically removed due to a storage space limit policy on the shared acquisition machine.

### Thiobarbituric acid reactive substances (TBAR) assay

Tissue/cellular lysates, containing 20 μg of proteins per sample, were analyzed using a commercial TBAR assay kit from Cayman Chemical (Ann Arbor, MI). This kit quantifies lipid peroxidation and malondialdehyde (MDA) contents via a colorimetric method. TBAR signal intensity was measured at 555 nm, with 595 nm as the reference wavelength. Mean values were calculated from five biological replicates.

### GSH/GSSG ratio

To measure the reduced glutathione (GSH) to oxidized glutathione (GSSG) ratio, a GSH/GSSG ratio kit from Thermo Fisher Invitrogen was employed. Briefly, cell or tissue lysates (20 μg of proteins per sample) were combined with 5,5’-dithiobis(2-nitrobenzoic acid) (DTNB), glutathione reductase, and NADPH (nicotinamide adenine dinucleotide phosphate). Subsequently, lysates were mixed with the reaction solution, and absorbance at 450 nm was monitored over five minutes using a spectrophotometer. A standard curve generated with GSH and GSSG standards allowed for quantification of their concentrations in the lysates. The ratio was normalized to protein concentration. Mean values were calculated from five biological replicates.

### SLC30A9 promoter luciferase activity assay

The anticipated PRDM1-binding region (*CTTTCTC*) or a mutated sequence (*CTAACTC*) based on the JASPAR database was strategically sub-cloned into a GV238 firefly luciferase vector [46]. Cervical cells were cultured to approximately 60% confluence, ensuring optimal conditions for transfection. The cells were then transfected with the *SLC30A9* promoter luciferase GV238 construct using Lipofectamine 3000 (Invitrogen, Shanghai, China). Following a 48-h incubation period, the firefly luciferase activity was assessed using a Glo luciferase reporter assay kit (Promega, Shanghai, China). *SLC30A9* promoter luciferase activity was also normalized to that of control cells. Mean values were calculated from five biological replicates.

### Chromatin immunoprecipitation (ChIP)

The ChIP assay was conducted following established protocols [46]. Briefly, tissue lysates were homogenized [47] and were subjected sonication to create fragmented genomic DNA. The DNA was then eluted from the beads, the formaldehyde cross-links were reversed by heating, and contaminating proteins were degraded by Proteinase K treatment. Immunoprecipitated (IP) was then performed using an anti-PRDM1 antibody to isolate the associated DNA. The eluted PRDM1-bound *SLC30A9 promoter DNA sequence* predicted by the JASPAR database was analyzed via quantitative PCR, with its level normalized to the control level. Two independent and verified pairs of primers were utilized: Primer Pair #1 and Primer Pair #2. Mean values were calculated from five biological replicates.

### ChIP assay in cultured cells

Cells were cross-linked with formaldehyde to covalently capture protein-DNA interactions, and following cell lysis, chromatin was extracted and mechanically fragmented by sonication. Chromatin fragments bound by histone H3 acetylated at lysine 27 (H3K27ac) were then selectively isolated through immunoprecipitation using a specific H3K27ac antibody (Cell Signaling Technology). These antibody-bound complexes were subsequently captured on magnetic beads and subjected to stringent washing steps. The DNA was then eluted from the beads, the formaldehyde cross-links were reversed by heating, and contaminating proteins were degraded by Proteinase K treatment. The released DNA fragments, representing regions enriched for H3K27ac, were then purified. Finally, quantitative PCR was performed using primers designed to amplify the target SLC30A9 gene locus by two different and verified primer pairs: Primer Pair #a and Primer Pair #b. Mean values were calculated from five biological replicates.

### Animal xenograft studies

Female nude mice (17.8–18.1 g) were procured from Shanghai SLAC Laboratory Animal Co., Ltd. Eight million genetically modified cervical cancer cells per mouse were subcutaneously (*s.c*.) implanted into their flanks. Tumor volume, body weight, and daily growth rate (mm³/day) were assessed 21 days post-inoculation, adhering to established protocols [37, 48]. Mice were randomly assigned to experimental groups using a random number generator prior to any treatment, which ensured an unbiased distribution of baseline characteristics. Furthermore, we implemented a double-blinding procedure where the researchers were unaware of the group assignments. Cages and images were coded to obscure group identities. Tumor sections underwent TUNEL staining followed by DAPI counterstaining for nuclear visualization. Immunohistochemistry (IHC) was performed using methods analogous to those employed for human tissues. The representative images presented were carefully selected to accurately reflect the average phenotype or trend observed within each group. All animal procedures were approved by the Institutional Animal Care and Use Committee (IACUC) and the Ethics Committee of the Affiliated Kunshan Hospital of Jiangsu University (#KY-2021-096-BR).

### Statistical analyses

Five biological replicates were utilized in all in vitro experiments. The results that displayed a normal distribution are represented as mean ± standard deviation (SD). To assess differences between two experimental groups, unpaired Student’s *t* tests were applied. In cases where three or more groups were compared, one-way ANOVA was performed, followed by post hoc multiple comparison tests, including Scheffé's and Tukey’s methods. A *P*value of less than 0.05 was considered statistically significant.

## Results

### Upregulation of SLC30A9 in cervical cancer tissues from local patients

**Fig. 1:**
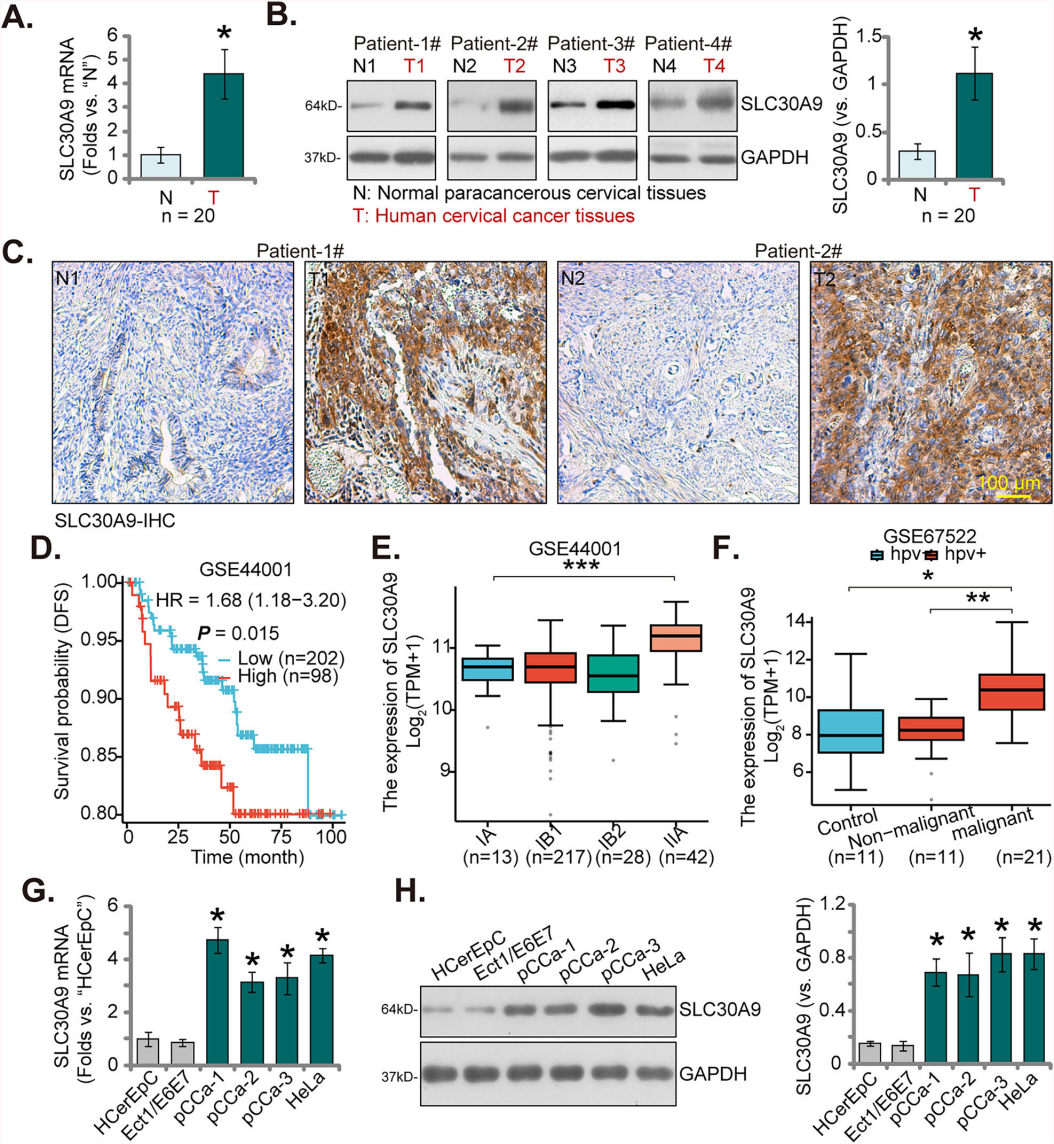
Upregulation of SLC30A9 in cervical cancer tissues from local patients. *SLC30A9* mRNA and protein expression in cervical cancer tissues (“T”) and corresponding normal cervical epithelial tissues (“N”) was analyzed from twenty primary cervical cancer patients *(n* = 20), and the results were quantified (**A** and **B**). Immunohistochemistry (IHC) images confirmed the upregulation of SLC30A9 protein in cervical cancer tissue samples from two representative patients (**C**). Kaplan-Meier curve showing the association between *SLC30A9* expression and disease-free survival (DFS) in 300 cervical cancer patients from the GSE44001 dataset (**D**). Box plot depicting the correlation between *SLC30A9* expression levels and FIGO stage in the GSE44001 dataset (**E**). Analysis of *SLC30A9* expression based on HPV status and tissue type using the GSE67522 dataset (**F**). *SLC30A9* mRNA and protein levels in the described cervical cancer cells and epithelial cells were tested (**G** and **H**, *n* = 5, biological repeats). Results are expressed as mean ± standard deviation (SD). Asterisks indicate significance with * *P* < 0.05 compared to the “N” tissues/ “HCerEpC” cells (**A**, **B**, **G**, and **H**). *** *P* < 0.001 (**E**); ** *P* < 0.01, * *P* < 0.05 (**F**). The experiments were repeated five times with similar results obtained. Scale bar = 100 μm.

We first investigated the expression of SLC30A9 in cervical cancer tissues from our local patients. Following the methodology outlined earlier [40], we collected cervical cancer samples (“T”) and corresponding paracancerous epithelial tissues (“N”) from twenty primary cervical cancer patients (*n* = 20). The analysis revealed that *SLC30A9* mRNA levels were significantly higher in the cancerous tissues (Fig. 1A). Additionally, the protein expression of SLC30A9 was markedly increased in the cervical cancer samples of four representative patients (Patients 1# to 4#) (Fig. 1B). Analyzing all twenty patient tissue Western blotting results, we found a significant upregulation of SLC30A9 protein in the cervical cancer tissues (Fig. 1B). Furthermore, immunohistochemistry (IHC) staining for Patients 1# to 2#’s tissue slides corroborated the pronounced increase in SLC30A9 protein levels in cervical cancer (Fig. 1C).

Transcriptomic data from 300 cervical cancer patients in the GSE44001 dataset were analyzed. The optimal expression threshold was determined using the surv_cutpoint function, and the results indicate that high *SLC30A9* expression is significantly associated with a worse disease-free survival (DFS) (Fig. 1D). Furthermore, a positive correlation was found between *SLC30A9* expression levels and higher FIGO (International Federation of Gynecology and Obstetrics) stages (Fig. 1E). Regarding the relationship with HPV genotype, analysis of the GSE67522 dataset revealed no significant difference in *SLC30A9* expression between HPV-negative and HPV-positive normal cervical tissues. However, its expression was found to be significantly upregulated in HPV-positive malignant tumors (Fig. 1F). These new findings from independent datasets provide more robust evidence for the prognostic significance of *SLC30A9* in cervical cancer and its potential association with tumor progression and HPV infection.

The expression of SLC30A9 in human cervical cancer cells was also examined. The findings revealed that both *SLC30A9* mRNA and protein levels were significantly increased in primary human cervical cancer cells (“pCCa-1,” “pCCa-2,” and “pCCa-3”, derived from three patients) and the immortalized HeLa cell line (Fig. 1G, H). In contrast, relatively low levels of *SLC30A9* mRNA and protein expression were noted in primary human cervical epithelial cells (HCerEpC) and the immortalized Ect1/E6E7 epithelial cell line (Fig. 1G, H). These results strongly indicate the overexpression of SLC30A9 in cervical cancer.

### Single-cell RNA-seq analysis reveals cell-type specific expression of *SLC30A9* and its association with mitochondrial function in cervical squamous cell carcinoma

**Fig. 2:**
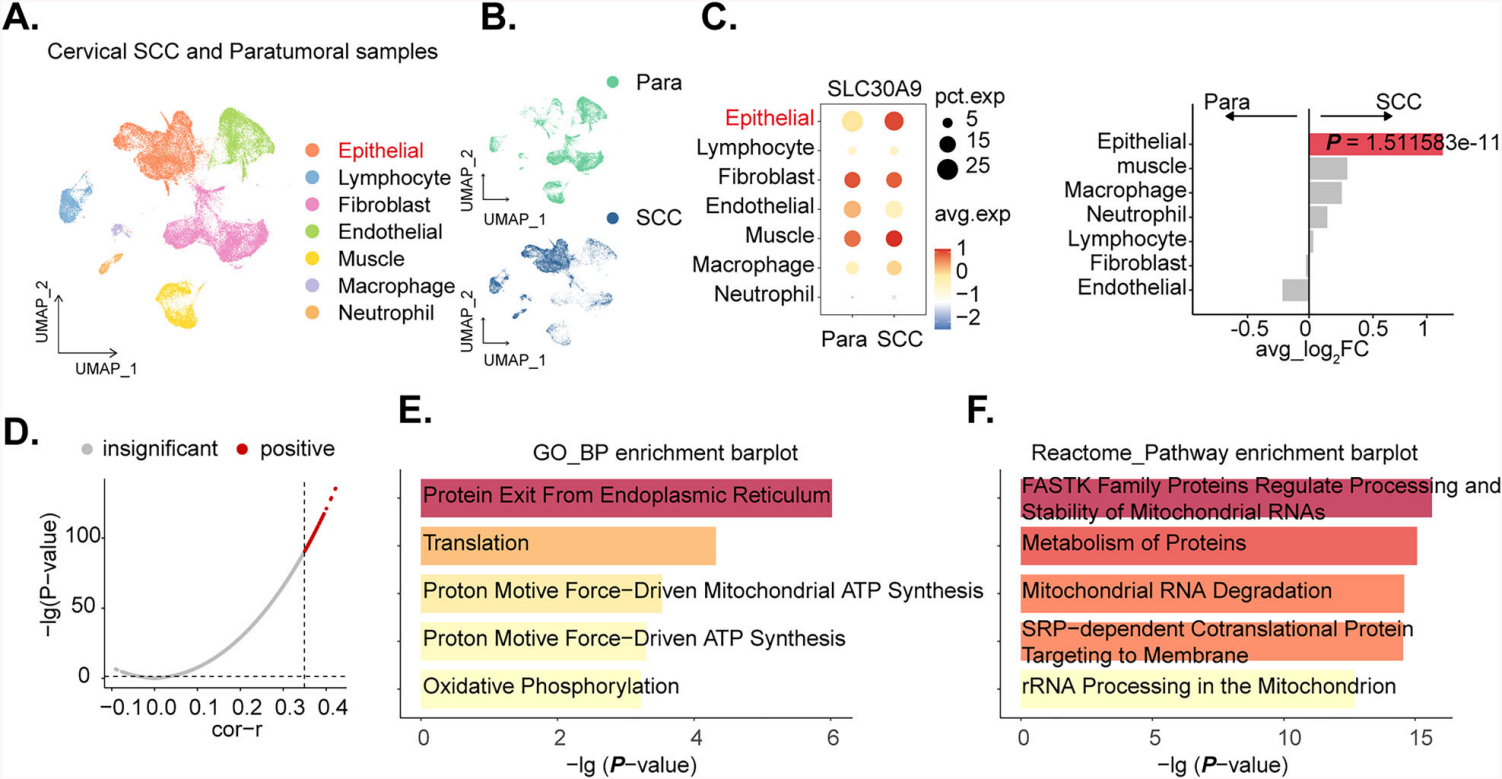
Single-cell RNA-seq analysis reveals cell-type specific expression of *SLC30A9* and its association with mitochondrial function in cervical squamous cell carcinoma. UMAP (Uniform Manifold Approximation and Projection) visualization of cervical squamous cell carcinoma (SCC) and paratumoral samples, colored by cell type, demonstrates distinct cellular heterogeneity within the tumor microenvironment (**A** and **B**). Dot plot illustrates the expression of *SLC30A9* across various cell types in cervical SCC and paratumoral samples, revealing significant upregulation in cervical SCC epithelial cells (**C**). Correlation plot showing genes positively correlated with *SLC30A9* in epithelial cells (**D**). Gene ontology (GO) biological process (BP) enrichment analysis of genes positively correlated with *SLC30A9* (**E**). Reactome pathway enrichment analysis of genes positively correlated with *SLC30A9* (**F**).

To gain insights into the cellular heterogeneity and molecular mechanisms underlying cervical squamous cell carcinoma (SCC), we performed a comprehensive analysis of single-cell RNA-seq data from the ArrayExpress database (E-MTAB). Dimensional reduction and clustering revealed distinct cell populations within the SCC and adjacent normal tissues (Fig. 2A, B). Cell types were annotated based on known marker genes and included epithelial cells, fibroblasts, endothelial cells, (smooth) muscle cells, and macrophages (Fig. 2A, B). Differential gene expression analysis identified *SLC30A9* as significantly upregulated in epithelial cells of the cervical SCC group compared to the adjacent normal tissue group (Fig. 2C). Although a minor increase in *SLC30A9* expression was observed in muscle and macrophages (*P* > 0.05, Fig. 2C), statistical analysis confirmed that only the *SLC30A9* expression within the epithelial cell population was significantly different between the tumor and para-tumoral tissues (Fig. 2C). To further investigate the potential role of *SLC30A9* in cervical SCC, we performed correlation analysis on epithelial cells. We identified the top 100 genes positively correlated with *SLC30A9* expression (Fig. 2D). Subsequent gene ontology and pathway enrichment analysis revealed significant enrichment in terms of protein translation, mitochondrial ATP synthesis, and oxidative phosphorylation (Fig. 2E). Furthermore, pathways related to metabolic proteins and mitochondrial RNA degradation were also enriched (Fig. 2F).

Re-evaluating the clustering at a resolution of 0.1 yielded 12 distinct cell clusters (Fig. S2A). To assign biological identities to these clusters, we examined the expression of known classical marker genes across all clusters. A dot plot summarizing the average expression and the percentage of cells expressing key markers within each cluster revealed distinct expression profiles (Fig. S2B). For instance, the epithelial cluster observed in Fig. 2A includes high-resolution clusters 2, 3, 7, and 8 in Figure S2B, showed high expression of epithelial markers such as *EPCAM*, *KRT5*, and *KRT17*. Endothelial markers (*PECAM1*, *CDH5*, *ENG*) were predominantly found in cluster 1 (Fig. S2B). Cluster 10 showed expression of neutrophil markers (*CSF3R*) (Fig. S2B). Feature plots for representative markers further confirmed these distinct expression patterns across the UMAP landscape (Fig. S2C), supporting the assignment of major cell types to the identified clusters.

### SLC30A9 silencing inhibits malignant behaviors of cervical cancer cells

**Fig. 3:**
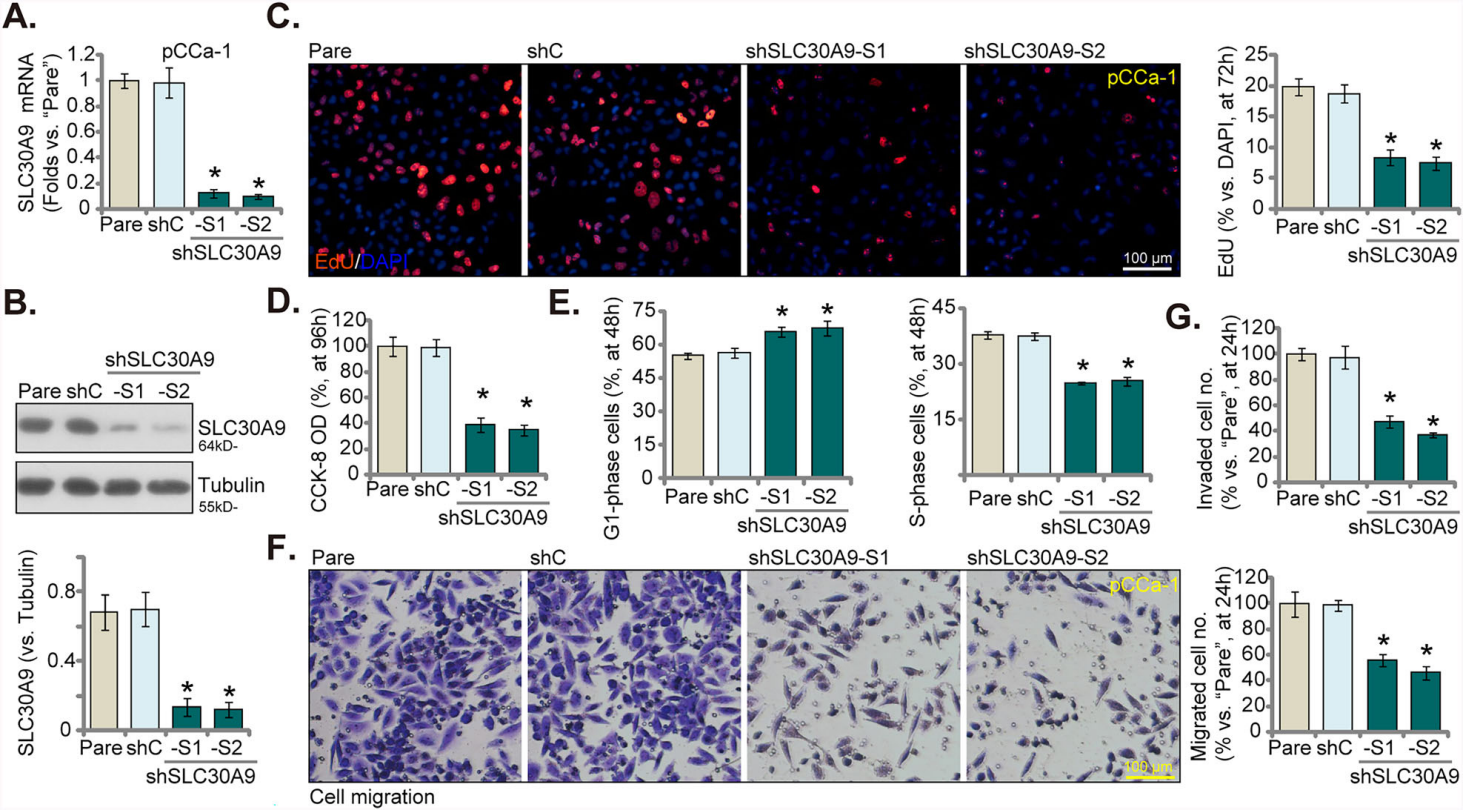
SLC30A9 silencing inhibits malignant behaviors of cervical cancer cells. The patient-derived primary human cervical cancer cells (pCCa-1) were transduced with either the lentiviral SLC30A9 shRNA (“shSLC30A9-S1/shSLC30A9-S2,” representing two distinct shRNAs) or a scrambled control shRNA (“shC”), stable cells were formed after puromycin selection. The expression levels of *SLC30A9* mRNA (**A**) and protein (**B**) were then assessed. Cells were further cultured in the basal medium for the applied time periods, cell proliferation (by measuring EdU-positive nuclei ratio, **C**), cell viability (CCK-8 OD, **D**), cell cycle progression (PI-FACS assays, **E**) as well as in vitro cell migration (“Transwell” assays, **F**) and invasion (results quantified in **G**) were tested, with results quantified. Time points at which these experiments were conducted were indicated on the Y-axis of the respective graphs. Results are expressed as mean ± standard deviation (SD, *n* = 5, biological repeats). “Pare” stands for the parental control cells. Asterisks indicate significance with * *P* < 0.05 compared to the “shC” treatment. The experiments were repeated five times with similar results obtained. Scale bar = 100 μm.

To explore the impact of overexpressed SLC30A9 on the aggressive behaviors of cervical cancer cells, we utilized a strategy involving shRNA-mediated knockdown to inhibit SLC30A9 expression. We transduced patient-derived primary human cervical cancer cells, identified as pCCa-1 [39, 40], with lentiviruses carrying two unique shRNA sequences directed against human *SLC30A9* (shSLC30A9-S1 and shSLC30A9-S2). Stable cells were then established. mRNA and protein detection occurred at the 6-h post-initiation of cell culture. Analyses using qRT-PCR and Western blotting confirmed a substantial decrease in both *SLC30A9* mRNA (Fig. 3A) and protein (Fig. 3B) levels in the stable pCCa-1 cells expressing the shSLC30A9 constructs. SLC30A9 shRNA significantly curtailed the proliferation of pCCa-1 cells and significantly decreased nuclear EdU incorporation (Fig. 3C). Additionally, silencing of SLC30A9 led to a marked reduction in cell viability, as assessed by the CCK-8 assay (Fig. 3D). Furthermore, it disrupted cell cycle progression, resulting in an increased percentage of cells in the G1 phase and a decrease in S-phase cells (Fig. 3E). Following this, we conducted Transwell migration and Matrigel invasion assays to evaluate how silencing of SLC30A9 could influence in vitro cell movement. The results indicated that the introduced shRNAs significantly hindered the migration and invasion capabilities of pCCa-1 cells in vitro (Fig. 3F, G, Fig. S3A).

We next expanded our study to include additional cervical cancer cell types. Specifically, we transduced primary cervical cancer cells from two other patients, designated as pCCa-2 and pCCa-3 [39, 40], along with the immortalized HeLa cell line, using lentivirus expressing shSLC30A9-S2. Stable cells were established through puromycin selection. qRT-PCR analysis demonstrated a significant reduction in *SLC30A9* mRNA levels across all three cervical cancer cell types (Fig. S3B). The knockdown of SLC30A9 led to a considerable decrease in cell proliferation, as indicated by EdU incorporation (Fig. S3C), as well as impaired in vitro cell migration (Fig. S3D) in both primary and immortalized cervical cancer cells. Additionally, the CCK-8 assay revealed decreased cell viability in the SLC30A9-silenced cervical cancer cells (Fig. S3E). These results reinforce the notion that SLC30A9 is crucial for promoting the malignant characteristics of cervical cancer cells.

In the experiments involving primary human cervical epithelial cells (HCerEpC) and the immortalized Ect1/E6E7 epithelial cell line [39, 40], we observed that treatment with shSLC30A9-S2 resulted in a remarkable reduction in *SLC30A9* mRNA expression levels (Fig. S3F). Yet when we evaluated cell proliferation using EdU staining assays (Fig. S3G), we found no substantial impact on the proliferative capacity of these non-cancerous cervical epithelial cells. These findings suggest that the effects of silencing SLC30A9 are likely unique to cancer cells, underscoring an important difference in the function of SLC30A9 between malignant and non-malignant cell behavior.

### SLC30A9 silencing induces apoptosis activation in cervical cancer cells

**Fig. 4:**
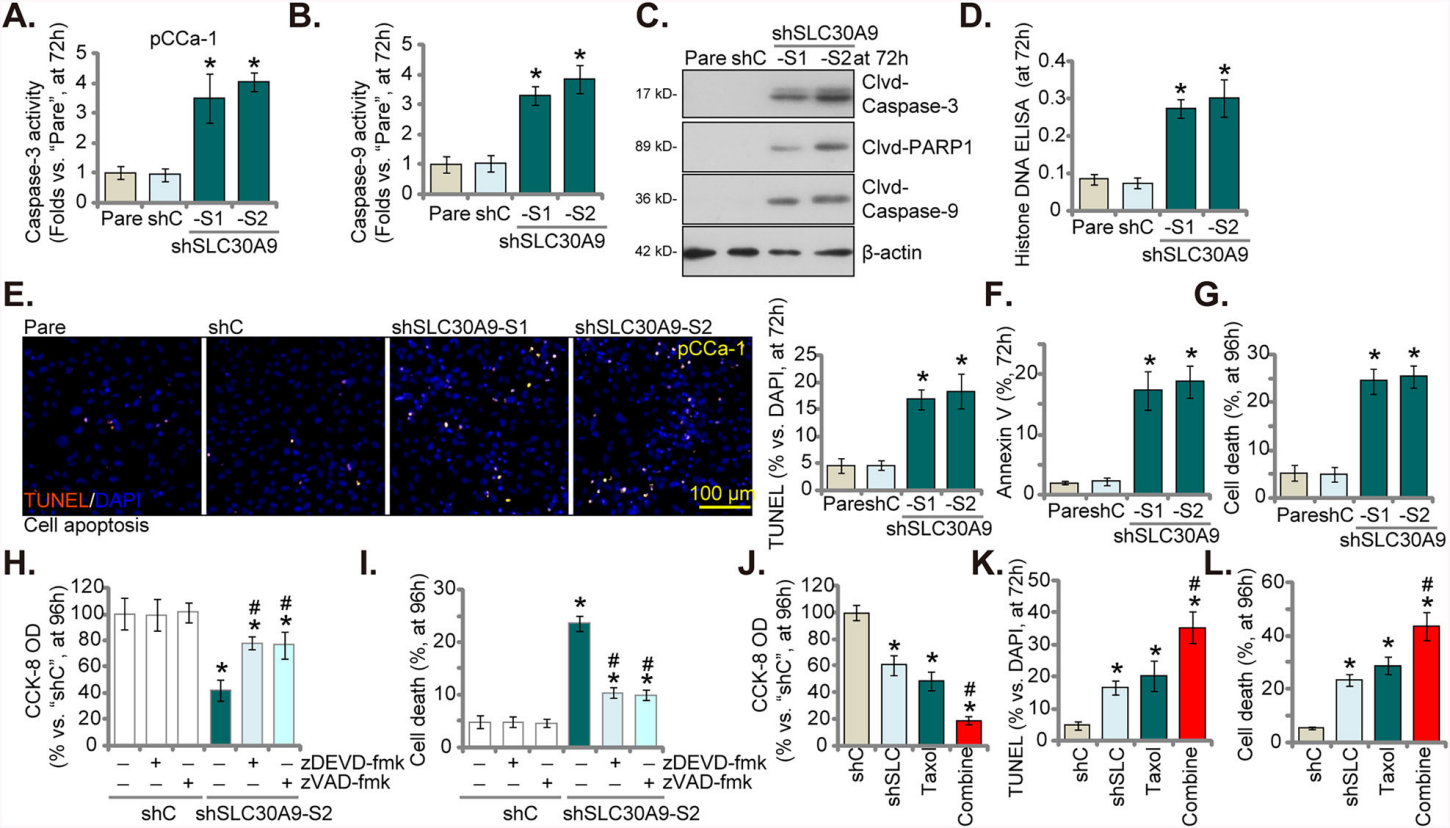
SLC30A9 silencing induces apoptosis activation in cervical cancer cells. The patient-derived primary human cervical cancer cells (pCCa-1) were transduced with either the lentiviral SLC30A9 shRNA (“shSLC30A9-S1/shSLC30A9-S2,” representing two distinct shRNAs) or a scrambled control shRNA (“shC”). Cells were further cultured in the basal medium for the applied time periods, the Caspase-3 activity (**A**), the Caspase-9 activity (**B**), expression levels of apoptosis-related proteins (**C**), and the Histone DNA contents (**D**) were tested, with cell apoptosis measured and quantified via the nuclear TUNEL staining (**E**) and the Annexin V FACS (**F**) assays. Cell death was tested via the Trypan blue staining assays (**G**). The shC- or shSLC30A9-S2-expressing pCCa-1 cells were incubated with z-DEVD-fmk (30 μM) or z-VAD-fmk (30 μM) for 96 h, cell viability and death were tested via CCK-8 (**H**) and Trypan blue staining (**I**) assays, respectively. pCCa-1 cells were allocated to different treatment conditions: shSLC30A9-S2 alone (shSLC), Paclitaxel (Taxol, 2 nM) alone, or a combination of both (designated the “Combine” group). A control group was treated with scramble control shRNA (“shC”). Following cultivation for designated time periods, cell viability was assessed using the CCK-8 assay (**J**), apoptosis was detected by nuclear TUNEL staining (**K**), and overall cell death was measured using the Trypan blue staining assay (**L**). Time points at which these experiments were conducted were indicated on the Y-axis of the respective graphs. Results are expressed as mean ± standard deviation (SD, *n* = 5, biological repeats). “Pare” stands for the parental control cells. Asterisks indicate significance with * *P* < 0.05 compared to the “shC” treatment. ^#^*P* < 0.05 vs. shSLC30A9-S2 only treatment (**H** and **I**). ^#^*P* < 0.05 vs. Taxol only treatment (**J**–**L**). The experiments were repeated five times with similar results obtained. Scale bar = 100 μm.

Silencing SLC30A9 via shRNA significantly impeded the viability, proliferation, cell cycle progression, and migratory capacity of primary/immortalized cervical cancer cells. Its influence on cellular apoptosis was examined. Primary pCCa-1 cells transfected with shSLC30A9-S1 or shSLC30A9-S2 exhibited a marked upregulation in both Caspase-3 and Caspase-9 activities (Fig. 4A, B). Moreover, SLC30A9 knockdown resulted in the proteolytic cleavage of Caspase-3, Caspase-9, and Poly (ADP-ribose) polymerase 1 (PARP1) in these cells (Fig. 4C). A concomitant increase in histone-bound DNA, an apoptotic marker, was observed in SLC30A9-shRNA-expressing pCCa-1 cells (Fig. 4D). This apoptotic cascade culminated in enhanced apoptosis in pCCa-1 cells, as evidenced by a substantial elevation in TUNEL-positive nuclei (Fig. 4E) and a higher proportion of Annexin V-positive pCCa-1 cells (Fig. 4F) at 72 h time point. Conversely, treatment with control shRNA (c-sh) did not induce apoptosis in pCCa-1 cells (Fig. 4A–F). Additional research demonstrated that the treatment of shSLC30A9-S1 or shSLC30A9-S2 resulted in a significant increase in cell death among pCCa-1 cells, as indicated by a higher proportion of cells exhibiting Trypan blue staining at 96 h time point (Fig. 4G). The application of the caspase-3 inhibitor z-DEVD-fmk or the broad-spectrum caspase inhibitor z-VAD-fmk significantly countered the decrease in viability and cell death triggered by shSLC30A9-S2 in pCCa-1 cells (Fig. 4H, I). Paclitaxel (Taxol) is a common chemotherapeutic drug for cervical cancer [49]. Our findings demonstrated that silencing the SLC30A9 gene in pCCa-1 cells, using shSLC30A9-S2 (shSLC), significantly potentiated Paclitaxel’s effects, leading to increased cell viability reduction (Fig. 4J), apoptosis (Fig. 4K), and overall cell death (Fig. 4L).

Furthermore, in additional primary/immortalized cervical cancer cells (pCCa-2, pCCa-3, and HeLa), the establishment of stable SLC30A9 knockdown using the shSLC30A9-S2-expressing lentiviral vector (as illustrated in Fig. 3) similarly led to Caspase-3 activation (Fig. S4A) and an augmented number of TUNEL-positive nuclei (Fig. S4B), thus corroborating the induction of apoptosis. Moreover, shSLC30A9-S2 also induced cell death in both primary and immortalized cervical cancer cells, as evidenced by increased Trypan blue staining (Fig. S4C). In contrast, in primary human cervical epithelial cells (HCerEpC) and the immortalized Ect1/E6E7 epithelial cell, SLC30A9 knockdown via shSLC30A9-S2 (see Fig. 3) did not significantly elevate Caspase-3 activity (Fig. S4D) or nuclear TUNEL staining (Fig. S4E), nor did it induce cell death (Fig. S4F), further supporting the cancer cell-specific effect of SLC30A9 knockdown. These findings collectively highlight the role of SLC30A9 in regulating apoptotic pathways within cervical cancer cells.

### SLC30A9 silencing results in considerable mitochondrial damage in cervical cancer cells

**Fig. 5:**
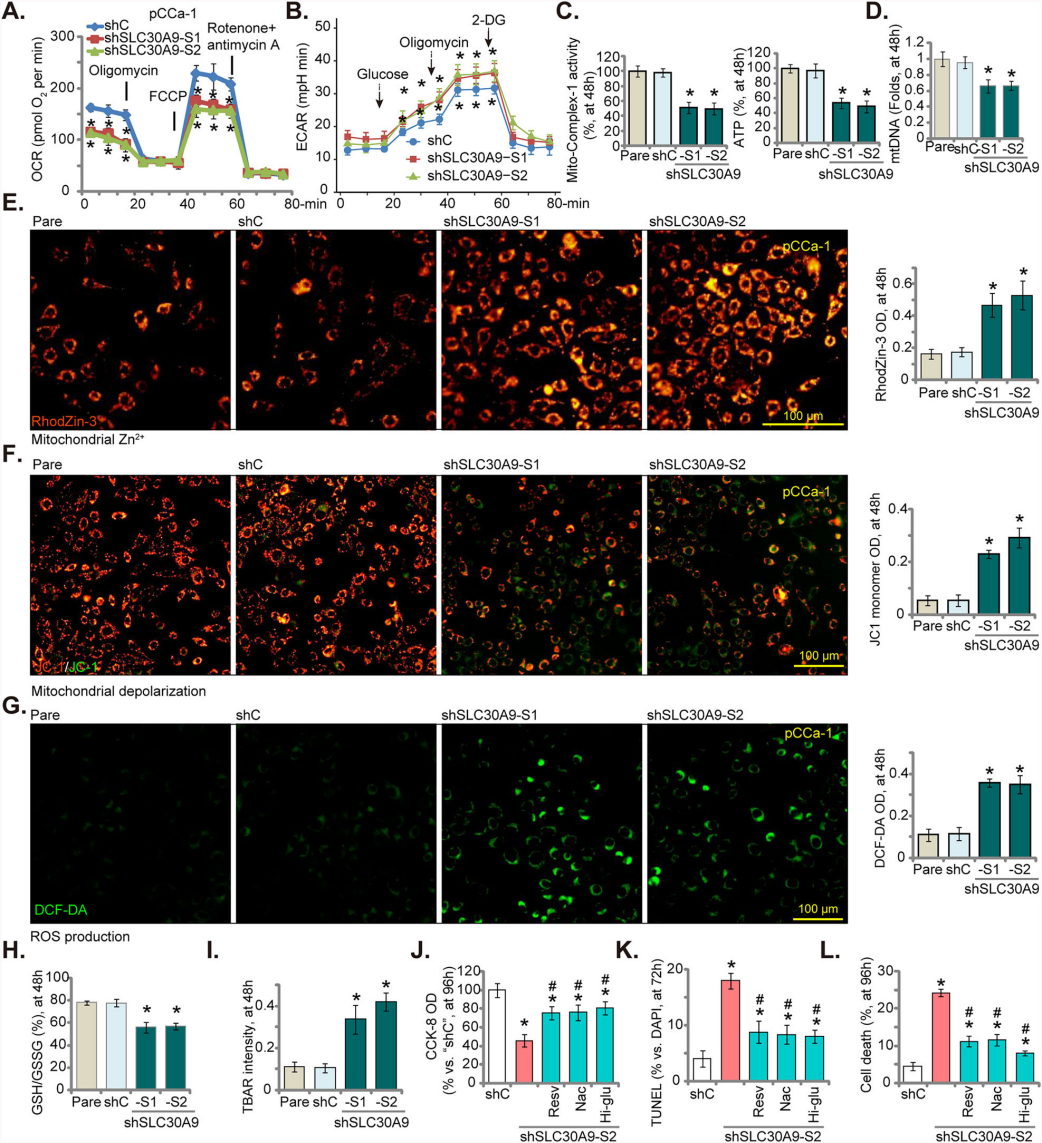
SLC30A9 silencing impairs mitochondrial function in cervical cancer cells. The pCCa-1 primary cells were transduced with either the lentiviral SLC30A9 shRNA (“shSLC30A9-S1/shSLC30A9-S2,” representing two distinct shRNAs) or a scrambled control shRNA (“shC”), stable cells were formed after puromycin selection. Cells were further cultured in the basal medium for the applied time periods, several key parameters were evaluated, including oxygen consumption rate (OCR) and extracellular acidification rate (ECAR) (measured using the Seahorse assay, **A** and **B**), mitochondrial complex I activity (**C**), ATP contents (**C**) and mtDNA contents (**D**); Mitochondrial Zn^2+^ levels (RhodZin-3 intensity) were measured (**E**). Mitochondrial depolarization was determined by JC-1 green monomer intensity (**F**), and ROS levels were measured via quantifying DCF-DA intensity (**G**). The GSH/GSSH ratio (**H**) and lipid peroxidation (measured using the TBAR assay, **I**) were also tested. The shSLC30A9-S2-expressing pCCa-1 cells were treated with N-acetylcysteine (NAC, 500 μM), resveratrol (Resv, 5 μM), or culture basal medium supplemented with an additional 7.5 mM glucose (Hi-glu). Following incubation for the indicated hours, cell viability was assessed using the CCK-8 assay (**J**), cell apoptosis measured by TUNEL staining (**K**), and overall cell death tested via Trypan blue exclusion (**L**). Time points at which these experiments were conducted were indicated on the Y-axis of the respective graphs. Results are expressed as mean ± standard deviation (SD, *n* = 5, biological repeats). “Pare” stands for the parental control cells. Asterisks indicate significance with * *P* < 0.05 compared to the “shC” treatment. ^#^*P* < 0.05 vs^.^ shSLC30A9-S2 only treatment (**J**–**L**). The experiments were repeated five times with similar results obtained. Scale bar = 100 μm.

We next aimed to investigate whether silencing SLC30A9, a zinc transporter within mitochondria, could impact mitochondrial functions in cervical cancer. Seahorse assay results indicated that the shRNA-induced knockdown of SLC30A9 significantly lowered both basal and maximal oxygen consumption rates (OCR) in pCCa-1 cells (Fig. 5A). We also measured the extracellular acidification rate (ECAR) to determine possible glycolytic compensation following SLC30A9 knockdown. The Seahorse analysis revealed that the knockdown of SLC30A9 in pCCa-1 cells led to a moderate but significant increase in both basal and glucose-stimulated ECAR compared to the control shC cells, indicating an enhanced glycolytic flux (Fig. 5B). Furthermore, a significant decline in mitochondrial complex I activity in cells with SLC30A9 knockdown was detected (Fig. 5C). This decline was associated with a reduction in ATP levels, as shown in Fig. 5C (the right panel), reinforcing the notion of inhibited mitochondrial respiration. The mtDNA contents were also significantly decreased (Fig. 5D). Levels of mitochondrial Zn^2+^ were significantly increased in SLC30A9-silenced pCCa-1 cells, as the RhodZin-3 signal was significantly enhanced (Fig. 5E). Thus, SLC30A9 silencing led to mitochondrial zinc accumulation in cervical cancer cells.

The knockdown of SLC30A9 also caused mitochondrial depolarization (Fig. 5F). In control cells, the uniform red fluorescence (JC-1 red aggregates) indicated a healthy, high mitochondrial membrane potential (MMP). In contrast, the mixed red and green signals (JC-1 green monomers) in the knockdown groups showed that the loss of SLC30A9 caused MMP depolarization that was not uniform across all cells (Fig. 5F). In SLC30A9-silenced pCCa-1 cells, there was a significant increase in reactive oxygen species (ROS) production, indicated by elevated DCF-DA green fluorescence intensity (Fig. 5G). Additionally, the GSH/GSSH ratio decreased in pCCa-1 cells expressing SLC30A9 shRNA (Fig. 5H). SLC30A9 silencing also led to a significant rise in TBAR activity in these cells, suggesting heightened lipid peroxidation (Fig. 5I). In contrast, treatment with c-sh did not adversely affect mitochondrial function in pCCa-1 cells (Fig. 5A–I). These results underscore the considerable mitochondrial damage resulting from SLC30A9 silencing in pCCa-1 primary cervical cancer cells.

N-acetylcysteine (NAC) is a well-characterized antioxidant, while Resveratrol (Resv) is known for its ability to activate signaling pathways that promote mitochondrial biogenesis [50–53]. Elevated glucose concentrations (Hi-glucose) in the culture medium can also enhance mitochondrial energy production. We evaluated the potential of these agents to counteract cellular damage in pCCa-1 cells expressing shSLC30A9-S2. The results demonstrate that treatment with NAC, Resv, or Hi-glucose significantly alleviated the detrimental cellular phenotypes observed. Specifically, these interventions effectively mitigated the shSLC30A9-S2-induced decrease in viability (CCK-8 OD, Fig. 5J), reduced cell apoptosis (TUNEL assays, Fig. 5K), and decreased overall cell death (Fig. 5L). Therefore, dysregulated mitochondrial function represents an important mechanism underlying the SLC30A9-silencing-induced anti-cervical cancer cell activity.

Early studies have shown that the absence of SLC30A9 resulted in an accumulation of zinc within the mitochondria [31]. This accumulation was significantly attenuated upon the simultaneous ablation of SLC25A25 (solute carrier family 25 member 25), and SLC25A25 is essential for mediating zinc import into the mitochondria [31]. Next, we silenced SLC25A25 using targeted siRNA in the shSLC30A9-S2 pCCa-1 cells (siSLC2525). Immunoblotting confirmed a significant reduction in SLC25A25 protein expression without affecting SLC30A9 in pCCa-1 cells (Fig. S5A). Importantly, SLC25A25 silencing successfully rescued the mitochondrial zinc accumulation phenotype; the RhodZin-3 signal was significantly reduced compared to the shSLC30A9-S2 pCCa-1 cells (Fig. S5B). This rescue was accompanied by an increase in ATP contents (Fig. S5C), a restoration of cell proliferation (tested by nuclear EdU incorporation, Fig. S5D), and an improvement in cell migration (Fig. S5E). The elevated cell death observed in the shSLC30A9-S2 pCCa-1 cells was also significantly mitigated by SLC25A25 silencing (Fig. S5F). These data indicate that SLC25A25 silencing can functionally compensate for the loss of SLC30A9 in regulating mitochondrial zinc levels and associated cellular processes.

To assess the consistency of SLC30A9 silencing in cervical cancer cells, we introduced shSLC30A9-S2 into additional primary/immortalized cancer cells (pCCa-2, pCCa-3, and HeLa). This knockdown significantly reduced mitochondrial complex I activity (Fig. S5G) and cellular ATP levels (Fig. S5H). The accumulation of JC-1 green fluorescent monomers indicated mitochondrial depolarization (Fig. S5I). These results highlight the critical role of SLC30A9 in maintaining mitochondrial function across different cervical cancer cells.

### SLC30A9 knockout demonstrates potent anti-cancer activity in primary cervical cancer cells

To confirm that the observed phenotypes above were specifically due to the loss of SLC30A9 and not off-target effects of the shRNA, we re-expressed an shRNA-resistant SLC30A9 cDNA (shR-SLC30A9) in the SLC30A9 knockdown pCCa-1 primary cancer cells (with shSLC30A9-S2). Western blot analysis demonstrated a significant increase in SLC30A9 protein levels in the re-expressed cells, indicating successful rescue (Fig. S6A). The re-expression of shRNA-resistant SLC30A9 successfully rescued the phenotypes associated with SLC30A9 loss. Compared to the shSLC30A9-S2 pCCa-1 cells, the shR-SLC30A9 cells exhibited a significant increase in ATP content (Fig. S6B), an increase in cell proliferation as measured by EdU incorporation (Fig. S6C), and a restoration of cell migration to levels comparable to the control cells (Fig. S6D). Furthermore, the increased cell death observed in the shSLC30A9-S2 pCCa-1 cells was significantly reduced upon re-expression of the resistant cDNA (Fig. S6E). These results confirm that the phenotypes observed following SLC30A9 knockdown were specifically caused by the loss of the SLC30A9 protein.

To further investigate the functional role of SLC30A9 in cervical cancer, we utilized the CRISPR/Cas9 gene editing technique to knock out (KO) SLC30A9 in cervical cancer cells. The pCCa-1 cells expressing Cas9 were transduced with a lenti-CRISPR/Cas9 vector that contained the sgRNA sequence targeting human *SLC30A9*: koSLC30A9-sg1 or koSLC30A9-sg2. Following puromycin selection and knockout verification, stable cells were established. In contrast to the control pCCa-1 cells that received the lenti-CRISPR/Cas9 empty vector with a nonsense sgRNA (“sgC”), the koSLC30A9 pCCa-1 cells exhibited a significant reduction in SLC30A9 protein levels (Fig. S6F, G). The mtDNA contents were also significantly decreased (Fig. S6H). The CRISPR/Cas9-mediated knockout of SLC30A9 led to mitochondrial depolarization (an increase in JC-1 green monomers, Fig. S6I). Additionally, both mitochondrial complex I activity (Fig. S6J) and cellular ATP levels (Fig. S6K) were significantly reduced in kSLC30A9 pCCa-1 cells, indicating impaired mitochondrial function. The knockout of SLC30A9 also suppressed the proliferation of pCCa-1 cells, decreasing CCK-8 cell viability (Fig. S6L) and the percentage of EdU-positive nuclei (Fig. S6M). Furthermore, in vitro migration of pCCa-1 cells was markedly inhibited after SLC30A9 knockout (Fig. S6N). The percentage of TUNEL-positive cells was elevated in kSLC30A9 pCCa-1 cells (Fig. S6O), indicating the activation of apoptosis. Thus, SLC30A9 knockout demonstrated significant anti-cancer effects in primary cervical cancer cells.

### SLC30A9 overexpression promotes the malignant phenotype of cervical cancer cells

**Fig. 6:**
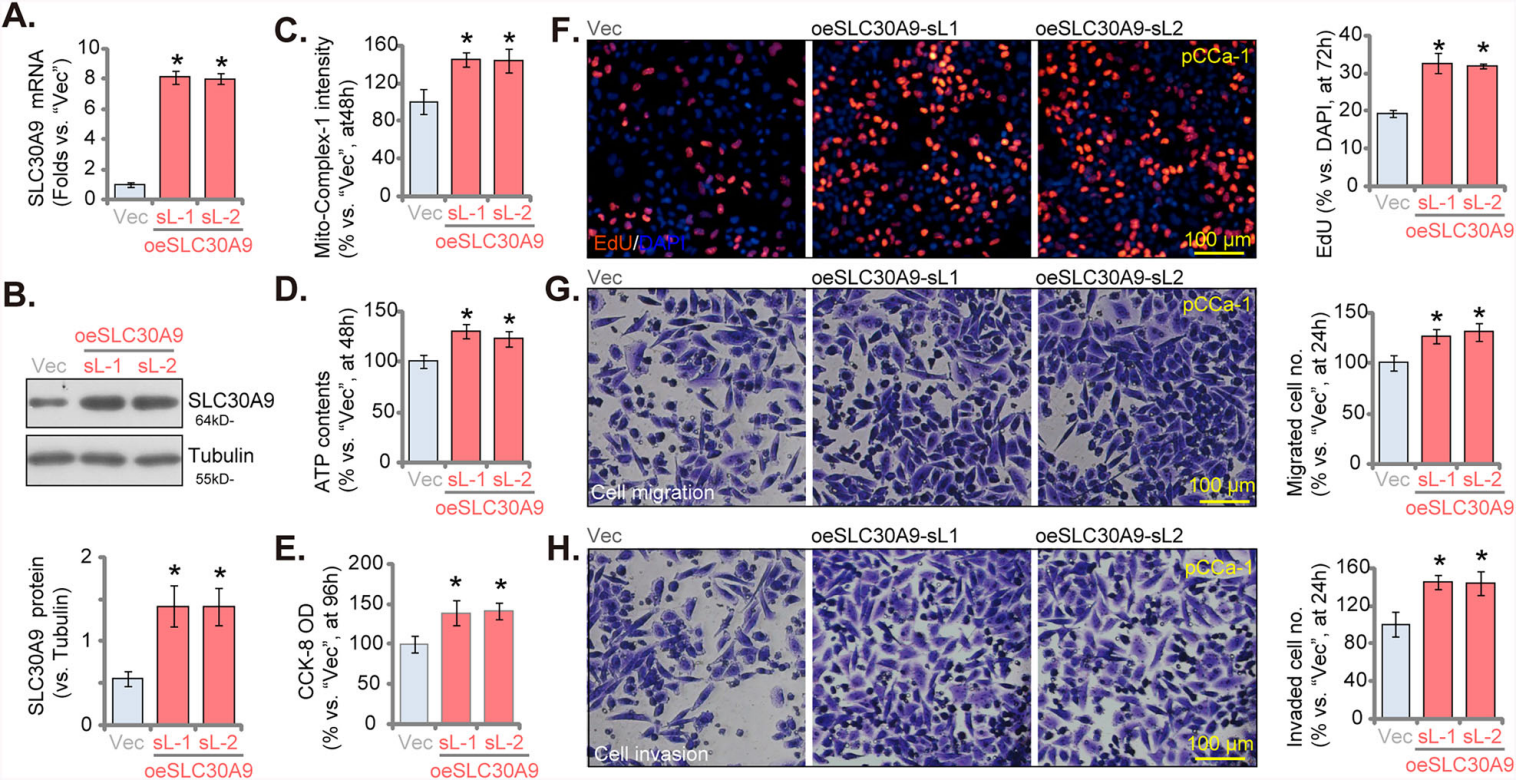
SLC30A9 overexpression promotes the malignant phenotype of cervical cancer cells. Primary pCCa-1 cells were transduced with a lentiviral construct expressing SLC30A9, generating two stable cell selections, oeSLC30A9-sL1 and oeSLC30A9-sL2, following selection and confirmation of overexpression. As a control, pCCa-1 cells were stably transduced with an empty vector (labeled “Vec”). The expression levels of SLC30A7 were subsequently assessed (**A** and **B**). Cells were further cultured in the basal medium for the applied time periods, mitochondrial complex I activity (**C**), ATP contents (**D**), and cell viability (CCK-8 assay, **E**) were measured. Cell proliferation (nuclear EdU incorporation, **F**), in vitro cell migration (“Transwell” assays, **G**) and invasion (“Matrigel Transwell” assays, **H**) were also tested. Time points at which these experiments were conducted were indicated on the Y-axis of the respective graphs. Results are expressed as mean ± standard deviation (SD, *n* = 5, biological repeats). Asterisks indicate significance with * ***P*** < 0.05 compared to the “shC” treatment. The experiments were repeated five times with similar results obtained. Scale bar = 100 μm.

Since SLC30A9 silencing or knocking out resulted in substantial anti-cancer effects in cervical cancer cells, we hypothesized that artificially overexpressing SLC30A9 could potentially promote malignant behaviors. We thus introduced a lentiviral SLC30A9 overexpression construct into pCCa-1 cells. After puromycin treatment, we established two stable cell selections: oeSLC30A9-sL1 and oeSLC30A9-sL2. In these oeSLC30A9 pCCa-1 cells, we observed a marked increase in both *SLC30A9* mRNA and protein levels (Fig. 6A, B), while SLC30A7 levels remained unchanged (Fig. 6A, B). SLC30A9 overexpression in pCCa-1 cells led to a significant upregulation of mitochondrial complex I activity (Fig. 6C) and ATP content (Fig. 6D). SLC30A9 overexpression enhanced cell viability, as measured by the CCK-8 assay (Fig. 6E). Moreover, oeSLC30A9 cells exhibited increased proliferation and a higher rate of nuclear EdU incorporation (Fig. 6F). Additionally, both the migratory (Fig. 6G) and invasive (Fig. 6H) capacities of pCCa-1 cells were significantly augmented following SLC30A9 overexpression.

We next extended our study to additional cervical cancer cells, including pCCa-2, pCCa-3 primary cells, and the immortalized HeLa cell line. These cells were transduced with the same lentivirus carrying the SLC30A9 overexpression construct. Stable cells, designated as oeSLC30A9, were generated through puromycin selection. qRT-PCR analysis demonstrated a significant upregulation of *SLC30A9* mRNA levels in all three cervical cancer cell types (Fig. S7A). Ectopic SLC30A9 overexpression led to increased ATP levels in the cervical cancer cells, suggesting enhanced mitochondrial function (Fig. S7B). Functionally, forced overexpression of SLC30A9 increased cell viability, as assessed by the CCK-8 assay (Fig. S7C). Furthermore, both proliferation (measured by nuclear EdU incorporation, Fig. S7D) and migration (measured by “Transwell” assays, Fig. S7E) were elevated in both primary and immortalized cervical cancer cells following SLC30A9 overexpression. Conversely, in the primary human cervical epithelial cells (HCerEpC) and the immortalized Ect1/E6E7 epithelial cell line, SLC30A9 overexpression, achieved using the same lentiviral construct (Fig. S7F), failed to significantly enhance cell viability (Fig. S7G) or proliferation (Fig. S7H). In conclusion, our findings indicate that SLC30A9 overexpression promotes the malignant phenotype of cervical cancer cells.

### PRDM1 functions as a key transcription factor for SLC30A9

We next conducted an analysis of the possible molecular mechanism underlying the elevated expression of SLC30A9 in cervical cancer. To identify potential transcriptional regulators of *SLC30A9*, we computationally predicted upstream transcription factors. The analyzed promoter region spanned 2000 bp upstream to 100 bp downstream of the transcription start site (TSS) of transcript ENST00000264451.12 (*hg38* chr4:41988530-41991000, + strand). Using four independent databases (UCSC-JASPAR, GTRD, GeneCard, CiiiDER), we identified four commonly predicted transcription factors: PRDM1, PRDM4, SP1, and KLF9 (Fig. S8A). Subsequent siRNA-mediated knockdown in pCCa-1 cervical cancer cells targeting these factors revealed that only PRDM1 silencing substantially downregulated *SLC30A9* mRNA expression (Fig. S8B).

Figure S8C provides a comprehensive visualization of the SLC30A9 genomic region using the UCSC Genome Browser, highlighting relevant features such as predicted transcription factor binding sites, histone modifications, and chromatin open regions. The predicted binding region for *PRDM1* is situated within a region exhibiting characteristics of a proximal enhancer-like element near the *SLC30A9* gene (Fig. S8C). This PRDM1 binding site shows a strong overlap with significant peaks of H3K27ac (Fig. S8C), suggesting that this specific locus likely possesses enhancer activity and possibly plays a role in the regulation of *SLC30A9*.

**Fig. 7:**
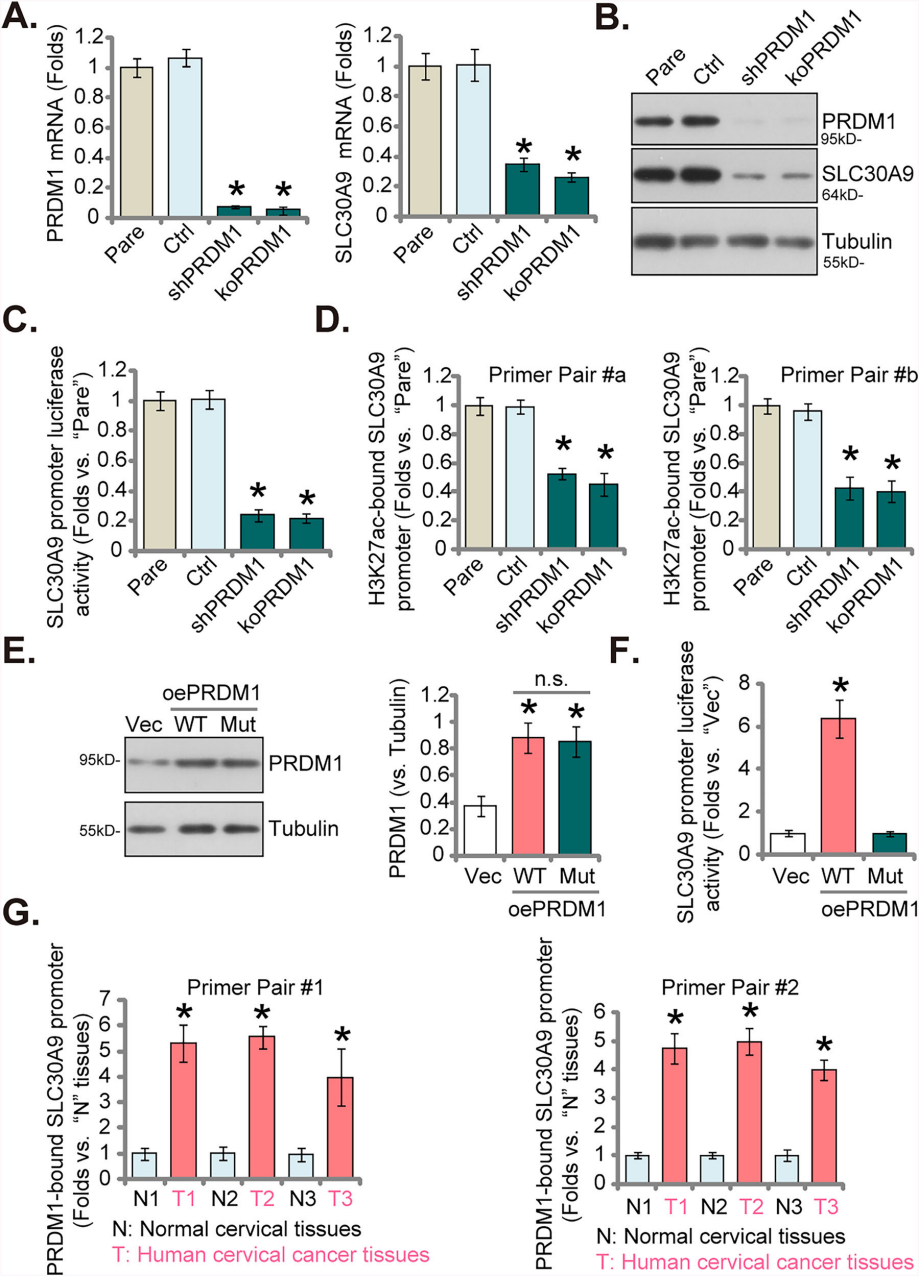
PRDM1 functions as a key transcription factor for SLC30A9. The pCCa-1 primary cancer cells were transduced with the lentiviral PRDM1 shRNA (“shPRDM1”); Alternatively, the Cas9-expressing pCCa-1 cells were transduced the lenti-CRISPR/Cas9 construct encoding sgRNA sequence against PRDM1 (“koPRDM1”). Control cells were transduced with the non-sense control shRNA plus the lenti-CRISPR/Cas9 construct with non-sense sgRNA (“Ctrl”). Stable cells were formed after puromycin selection, expression of listed genes and proteins was shown (**B** and **C**); The *SLC30A9* promoter luciferase activity was analyzed and results were quantified (**D**). pCCa-1 cells were co-transfected with a PRDM1 overexpression plasmid and either a wild-type (wt) or mutant (mut) SLC30A9 promoter luciferase reporter construct. The mutant construct contains a point mutation in the predicted PRDM1-binding site (*CTTTCT*C → *CTAACTC*). Control cells were with the empty vector (“Vec”). Expression of listed proteins was shown (**E**); Luciferase reporter was also tested and results were quantified (**F**). Chromosome IP (ChIP) showed the relative levels of the SLC30A9 promoter binding to the PRDM1 protein in the listed human tissues (**G**). Time points at which these experiments were conducted were indicated on the Y-axis of the respective graphs. Results are expressed as mean ± standard deviation (SD, *n* = 5, biological repeats). “Pare” stands for the parental control cells. Asterisks indicate significance with * ***P*** < 0.05 compared to the “Ctrl” cells, “Vec” cells, or “N” tissues. The experiments were repeated five times with similar results obtained.

Using the same viral shRNA and CRISPR/Cas9 techniques, we were able to successfully knock down and knock out PRDM1 in primary cultured pCCa-1 cervical cancer cells (Fig. 7A, B). PRDM1 silencing or KO led to a marked decrease in both *SLC30A9* mRNA and protein expression levels (Fig. 7A, B) in pCCa-1 primary cervical cancer cells, accompanied by a significant reduction in the luciferase activity of the *SLC30A9*’s promoter (Fig. 7C).

We also conducted chromatin immunoprecipitation (ChIP) analysis for H3K27ac using two distinct pairs of primers (Primer Pair #a and Primer Pair #b). This analysis revealed a significant decrease in H3K27ac enrichment at the SLC30A9 promoter region following PRDM1 knockdown or knockout (Fig. 7D). Therefore, PRDM1 could possibly function as a positive regulator of *SLC30A9* gene expression, potentially by recruiting or stabilizing the machinery responsible for H3K27 acetylation at this specific genomic location.

Overexpression of PRDM1 (Fig. 7E) in pCCa-1 cells significantly increased SLC30A9 promoter luciferase activity of a reporter construct containing the wild-type PRDM1-binding site (wt) (Fig. 7F). In contrast, this activation was abolished when a reporter construct containing point mutations in the predicted PRDM1-binding site (CTTTCTC → CTAACTC) was used (Fig. 7F). These results demonstrate that the specific CTTTCTC motif is essential for PRDM1-mediated transcriptional activation of SLC30A9.

Furthermore, ChIP analysis in human tissues, using two distant primer pairs (Primer Pair #1 and Primer Pair #2), demonstrated that the binding activity of PRDM1 to the *SLC30A9* promoter region was significantly elevated in cervical cancer tissues compared to adjacent normal cervical tissues (Fig. 7G). These findings suggest that PRDM1’s enhanced binding to the promoter region possibly facilitating increased transcription and contributing to the aberrant overexpression of SLC30A9 in cervical cancer.

### SLC30A9 silencing inhibits cervical cancer xenograft growth in nude mice

**Fig. 8:**
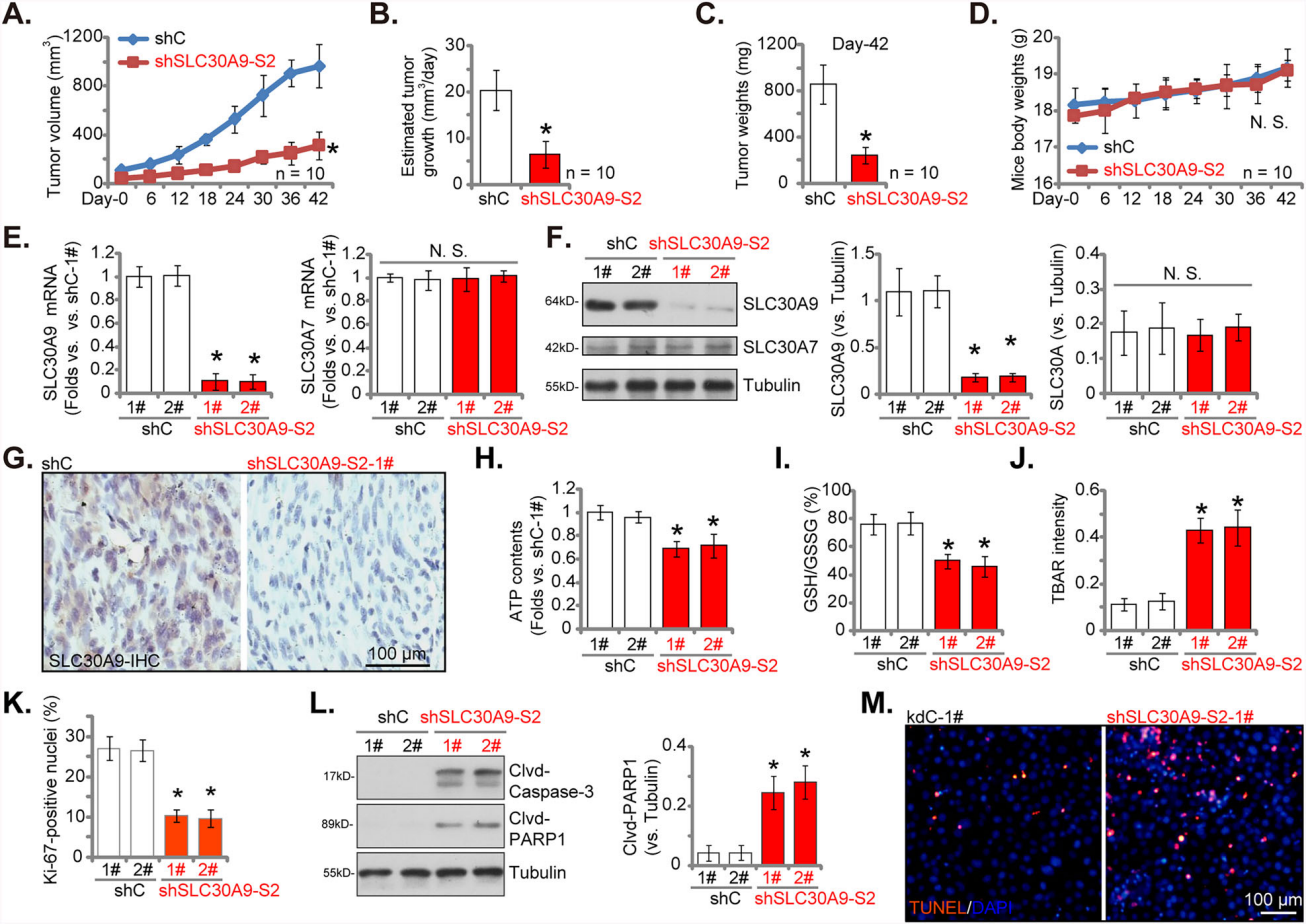
SLC30A9 silencing inhibits cervical cancer xenograft growth in nude mice. At eight million cells per mouse, pCCa-1 cervical cancer cells stably expressing SLC30A9 shRNA (shSLC30A9-S2) or control shRNA (shC) were subcutaneously inoculated into female nude mice. Ten mice were included in each experimental cohort. Tumor growth was monitored for 42 days, with tumor volume (**A**) and body weight of the animals (**D**) assessed every six days commencing on day 21 post-injection, designated as “Day-0”. Daily tumor growth rates were calculated (**B**). At day 42, mice were euthanized, and tumors were excised and weighed (**C**). Two xenografts per group (labeled 1# and 2#) were selected for further analysis. Tissue lysates were prepared for the assessment of SLC30A9 and SLC30A7 expression (**E** and **F**), as well as cleaved-caspase-3 and cleaved-PARP1 levels (**L**). ATP contents (**H**), the GSH/GSSG ratio (**I**), and TBAR intensity (**J**) were also quantified in the xenograft tissue lysates. Immunohistochemistry (IHC) was performed on tumor sections to evaluate SLC30A9 protein expression (**G**) and quantify Ki-67-positive nuclei ratio (**K**). Furthermore, TUNEL/DAPI staining was conducted on xenograft sections to assess apoptosis (**M**). Results are expressed as mean ± standard deviation (SD). Asterisks indicate significance with * ***P*** < 0.05 compared to the “shC” group. “N.S.” denotes no statistically significant difference (***P*** > 0.05). For analyses pertaining to tumor volume (**A**), tumor growth rate (**B**), tumor weight (**C**), and mouse body weight (**D**), data were derived from ten mice per group (*n* = 10). For analyses of panels **E**–**M**, each xenograft was dissected into five segments (*n* = 5) for individual analysis. Scale bar = 100 μm.

To explore the functional impact of SLC30A9 on the in vivo proliferation of cervical cancer cells, we developed subcutaneous xenograft models in nude mice using pCCa-1 cells that were engineered to express either shSLC30A9-S2 (see Figs. 3–5) or a control shRNA (shC). Tumor volume was assessed 21 days post-cell injection (designated as “Day-0”) and tracked over a 42-day duration. We observed a notable decrease in tumor growth within the shSLC30A9-S2 group when compared to the control pCCa-1 xenografts (Fig. 8A). Daily measurements of xenograft growth further validated this observation, indicating a significant inhibition of xenograft expansion in the shSLC30A9-S2 group (Fig. 8B). By the conclusion of the study (“Day-42”), the shSLC30A9-S2 xenografts showed considerably lower tumor weights relative to the control xenografts (Fig. 8C). Throughout the experiment, body weight remained stable across both groups (Fig. 8D). To gain deeper insights into the mechanisms, we evaluated SLC30A9 expression in the excised xenografts, collecting two samples from each group at Day-42. qRT-PCR analysis revealed a substantial decrease in *SLC30A9* mRNA levels in the shSLC30A9-S2 pCCa-1 xenografts (Fig. 8E), alongside a significant reduction in SLC30A9 protein levels (Fig. 8F). The expression of the control SLC30A7 showed no significant variation (Fig. 8E, F). IHC staining in tumor sections further confirmed the effective knockdown of SLC30A9 protein expression within the shSLC30A9-S2 pCCa-1 xenografts (Fig. 8G).

In the shSLC30A9-S2 pCCa-1 xenograft tissues, we observed signs of mitochondrial impairment, characterized by reduced ATP levels (Fig. 8H). Furthermore, the GSH/GSSG ratio was significantly lower in tumors where SLC30A9 was silenced (Fig. 8I). Enhanced TBAR intensity indicated heightened lipid peroxidation and oxidative stress in the pCCa-1 xenograft tissues after SLC30A9 knockdown (Fig. 8J). Xenografts of pCCa-1 cells with silenced SLC30A9 (shSLC30A9-S2) also exhibited a marked reduction in Ki-67-positive cells compared to controls (Fig. 8K), signifying a substantial suppression of tumor growth in vivo. This anti-proliferative effect was accompanied by heightened apoptosis. Western blotting analysis revealed elevated levels of cleaved-caspase-3 and cleaved-PARP1 in shSLC30A9-S2 xenograft tissues (Fig. 8L). Furthermore, nuclear TUNEL fluorescence staining provided corroborative evidence of apoptosis, demonstrating a significantly higher proportion of apoptotic nuclei in shSLC30A9-S2 xenografts (Fig. 8M). These findings collectively underscore that silencing SLC30A9 effectively inhibited mitochondrial function, induced apoptosis and suppressed tumor growth of pCCa-1 xenografts i*n vivo*.

## Discussion

Mitochondrial hyperfunction plays an important role in the progression of cervical cancer by influencing cellular metabolism, apoptosis, and other mechanisms [17, 27, 54–56]. In cervical cancer cells, enhanced mitochondrial respiration facilitates ATP generation, which supports the biosynthetic demands of rapidly proliferating cancer cells [27, 54–56]. This metabolic reprogramming can also lead to genomic instability and activate oncogenic signaling pathways. An elevated level of mitochondrial DNA copy number in exfoliated cervical cells from HPV-positive women demonstrated a significant association with the development of cervical cancer [54]. Furthermore, altered mitochondrial dynamics may disrupt apoptosis, allowing cancer cells to evade programmed cell death, thereby enhancing tumor survival and progression [17, 27, 54–56]. The interplay between mitochondrial hyperfunction and the tumor microenvironment also fosters a conducive setting for tumorigenesis through the modulation of immune responses [18–22]. Consequently, targeting mitochondrial function presents a promising avenue for therapeutic interventions in cervical cancer, highlighting the need for further exploration of mitochondrial dynamics in oncogenesis [18–22]. Atovaquone, by inhibiting mitochondrial complex III, selectively targeted cervical cancer cells with high mitochondrial biogenesis, demonstrating its potential as a novel therapeutic for these cancers [55].

Differential expression of zinc-exporters, specifically the SLC30A protein family, is associated with prostate cancer progression [57], suggesting these proteins could be new targets for therapeutic intervention [57]. Our findings provide robust evidence for SLC30A9 as a promising therapeutic target of cervical cancer, supported by a range of analyses. Elevated levels of SLC30A9 were observed in cervical cancer tissues from patients undergoing local treatment, as well as in several primary and established cervical cancer cells. Single-cell RNA sequencing revealed that *SLC30A9* is markedly overexpressed in the malignant epithelial cell population of cervical squamous cell carcinoma. Functional assays indicated that silencing SLC30A9 through shRNA or knockout using CRISPR/Cas9 method significantly hindered the viability, proliferation, and migratory abilities of cervical cancer cells, while simultaneously triggering apoptotic pathways. In contrast, overexpressing SLC30A9 using a lentiviral construct exacerbated malignant characteristics. Furthermore, SLC30A9 knockdown led to a remarkable decrease in the growth of cervical cancer xenografts, further emphasizing its potential as a valuable target for therapeutic strategies in the treatment of cervical cancer.

Our findings strongly suggest that SLC30A9 overexpression plays a pivotal role in driving the hyperfunction of cervical cancer cells. We observed that silencing SLC30A9 through shRNA or genetic knockout significantly disrupted mitochondrial function. This manifested as inhibited mitochondrial respiration, reduced mtDNA contents, decreased mitochondrial membrane potential, and impaired activity of mitochondrial complex I, leading to reduced ATP production. The ensuing energy deficit triggered an increase in ROS production, culminating in oxidative injury. The antioxidant NAC, Resveratrol (Resv), and elevated glucose concentrations (Hi-glucose) significantly mitigated the reduced viability, increased apoptosis, and overall cell death induced by shSLC30A9-S2 in primary cervical cancer cells. Conversely, overexpression of SLC30A9 in various cervical cancer cells demonstrated enhanced mitochondrial complex I activity and ATP production. These findings were further corroborated in vivo, as SLC30A9-silenced cervical cancer xenograft tissues exhibited decreased ATP production and increased oxidative injury. Collectively, these results highlight the critical role of overexpressed SLC30A9 in maintaining mitochondrial hyperfunction in cervical cancer cells.

Our findings indicate that silencing or knockout the zinc transporter SLC30A9 in pCCa-1 cervical cancer cells leads to a significant increase in mitochondrial zinc (Zn ^2+^) levels, a result consistent with previous research [31]. This accumulation of mitochondrial zinc is detrimental to cell function. We were able to functionally rescue this phenotype by simultaneously silencing SLC25A25, a mitochondrial carrier protein known to be essential for zinc import into the mitochondria [31]. The attenuation of mitochondrial zinc accumulation following SLC25A25 knockdown not only restored ATP levels but also improved cell proliferation and migration while significantly mitigating cell death. These results collectively suggest that the toxic effects of SLC30A9 loss are directly linked to the excessive influx of zinc into the mitochondria, a process likely mediated by SLC25A25.

The precise mechanism by which SLC30A9-mediated mitochondrial zinc (Zn^2+^) homeostasis might contribute to mitochondrial hyperfunction and the malignant phenotype remains to be elucidated, though several putative pathways can be considered. One plausible hypothesis focuses on the zinc-dependent proteases OMA1 metalloendopeptidase and YME1L (YME1 like 1 ATPase), which are known to regulate OPA1 (optic atrophy 1), a key protein in inner mitochondrial membrane fusion [31, 58–60]. It is possible that SLC30A9 overexpression leads to a depletion of zinc within the mitochondrial matrix. Such a depletion could foreseeably inhibit OMA1/YME1L activity (and possible other matrix proteases), which in turn might promote the accumulation of fusion-competent, long-form OPA1 and subsequently drive the mitochondrial fusion [31, 58–60]. It is also possible that the metabolic reprogramming in these cells is linked to mitochondrial aconitase, a key TCA cycle enzyme that can be inhibited by zinc [61]. A hypothetical SLC30A9-mediated depletion of mitochondrial zinc could relieve this inhibition, potentially enhancing aconitase activity to boost the TCA cycle [61]. Such a mechanism would be consistent with our observation that glucose supplementation partially rescued the viability and inhibited apoptosis in SLC30A9-knockdown cells. These possibilities provide a compelling framework for future investigations into this SLC30A9-mediated zinc-dependent regulatory axis in cervical cancer cells.

This study delves into the molecular mechanisms driving SLC30A9 overexpression in cervical cancer. Bioinformatics analysis pinpointed PRDM1 as a potential transcription factor with high affinity for the *SLC30A9* gene. Functional experiments demonstrated a significant reduction in SLC30A9 expression at both the mRNA and protein levels following PRDM1 silencing or KO in primary cervical cancer cells. Concomitantly, PRDM1 depletion resulted in decreased luciferase activity driven by the SLC30A9 promoter in primary cervical cancer cells, further supporting the role of PRDM1 in regulating SLC30A9 transcription. Importantly, ChIP assays revealed significantly increased binding of PRDM1 to the SLC30A9 promoter region in cervical cancer tissues, providing strong evidence for PRDM1’s direct involvement in upregulating SLC30A9 expression. These findings collectively suggest that PRDM1 acts as a crucial transcription factor for SLC30A9 in cervical cancer, with its enhanced binding to the promoter region likely driving the aberrant overexpression of SLC30A9 and potentially contributing to the pathogenesis of this malignancy.

## Supplementary information

Fig S1-S8

## Data Availability

All data are available in the Figures and supplementary files.
