# Supplementary material for: PRDM1-driven SLC30A9 overexpression contributes to the malignant phenotype of cervical cancer cells via promoting mitochondrial hyperfunction
Source: Cell Death Dis. 2025 Dec 19;16(1):895. doi: 10.1038/s41419-025-08264-x (PMC12717079; doi:10.1038/s41419-025-08264-x)
Supplement: Supplementary file 1 — Fig S1-S8 [file 41419_2025_8264_MOESM1_ESM.pdf]

Figure S1.

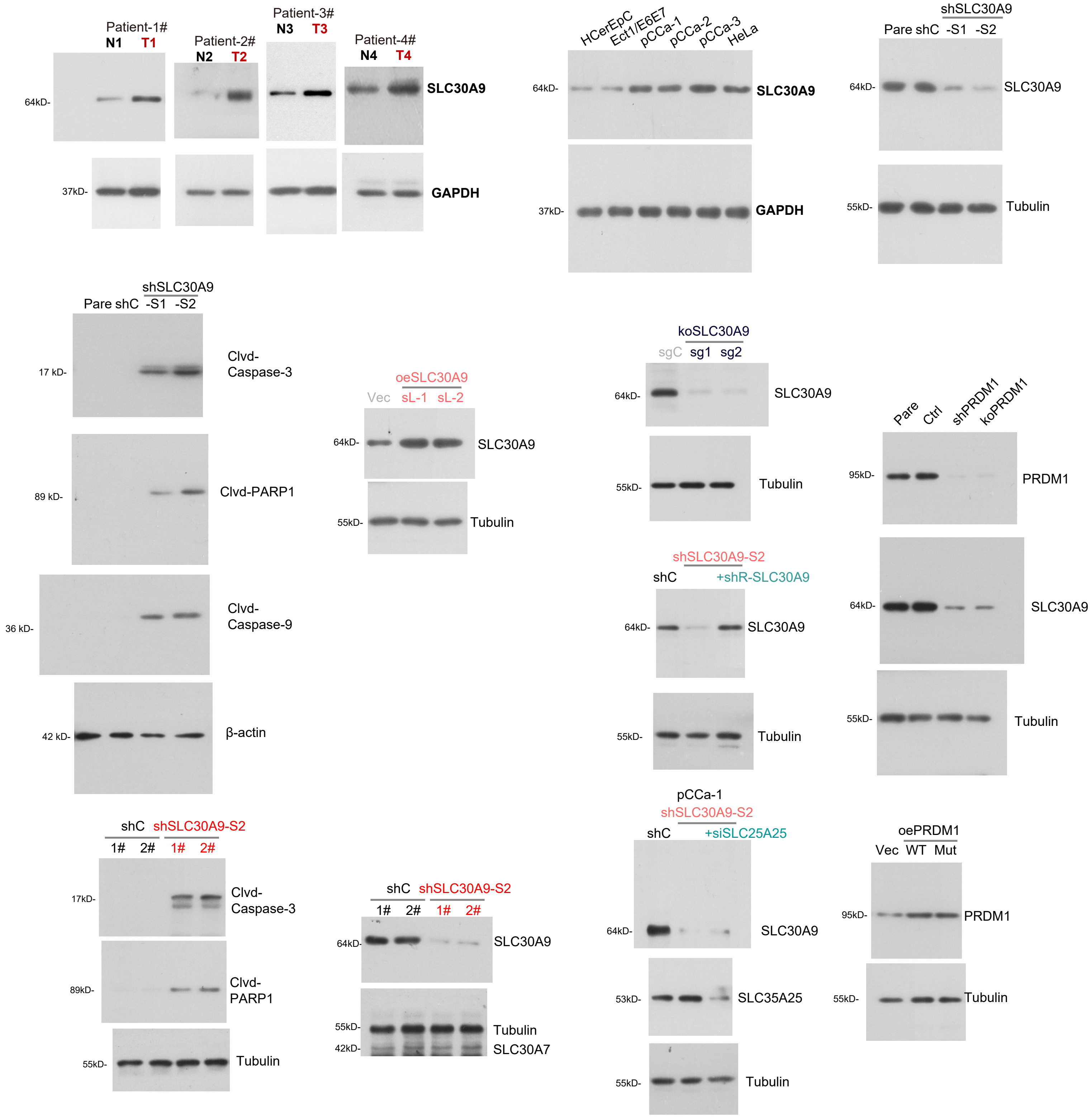

Figure S1. The uncropped blotting images of the study.

Figure S2.

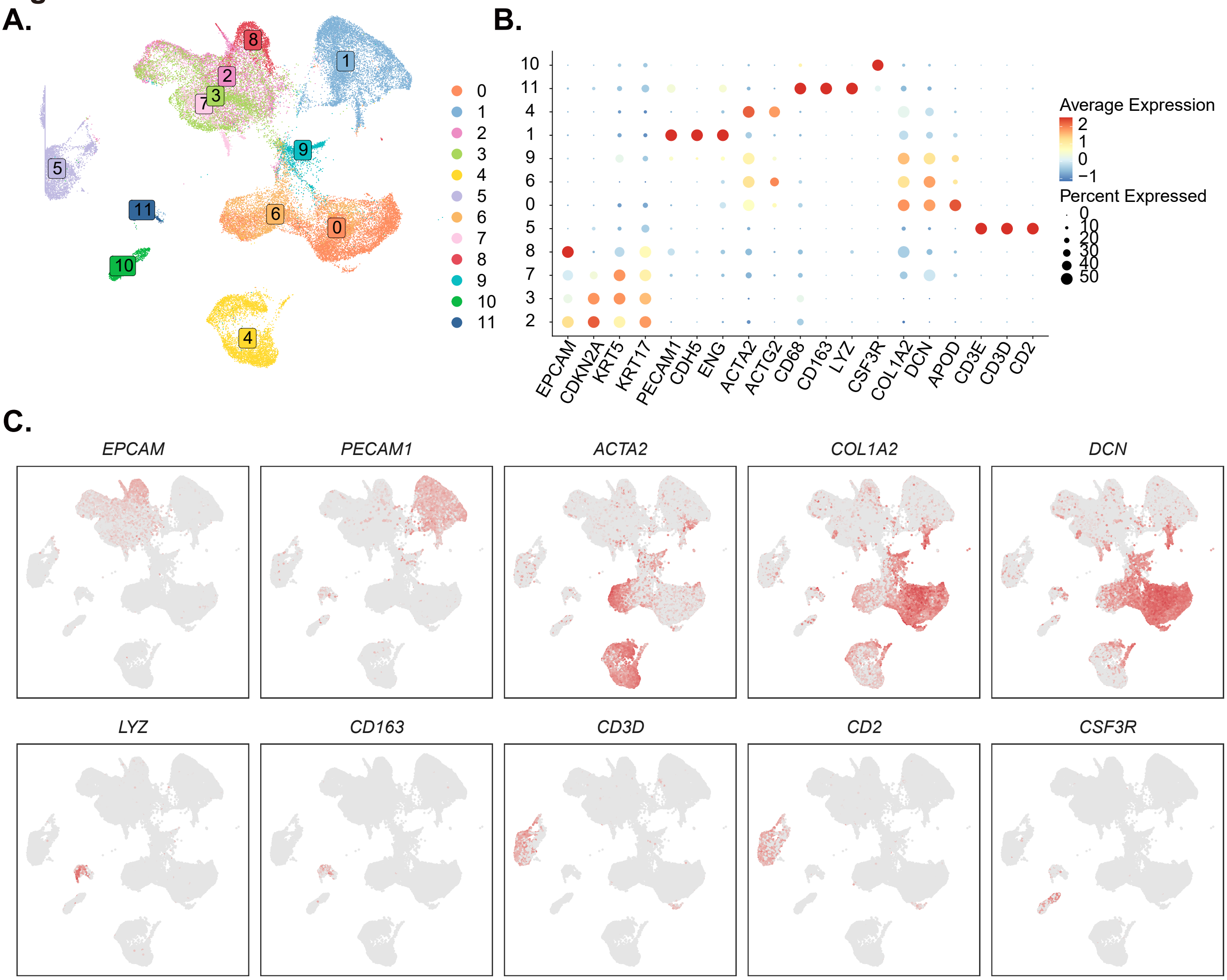
A.
0
1
2
3
4
5
6
7
8
9
10
11
B.
EPCAM
CDKN2A
KRT5
KRT17
PECAM1
CDH5
ENG
ACTA2
ACTG2
CD68
CD163
LYZ
CSF3R
COL1A2
DCN
APOD
CD3E
CD3D
CD2
Average Expression
2
1
0
−1
Percent Expressed
0
10
20
30
40
50
C.
EPCAM
PECAM1
ACTA2
COL1A2
DCN
LYZ
CD163
CD3D
CD2
CSF3R

Figure S3

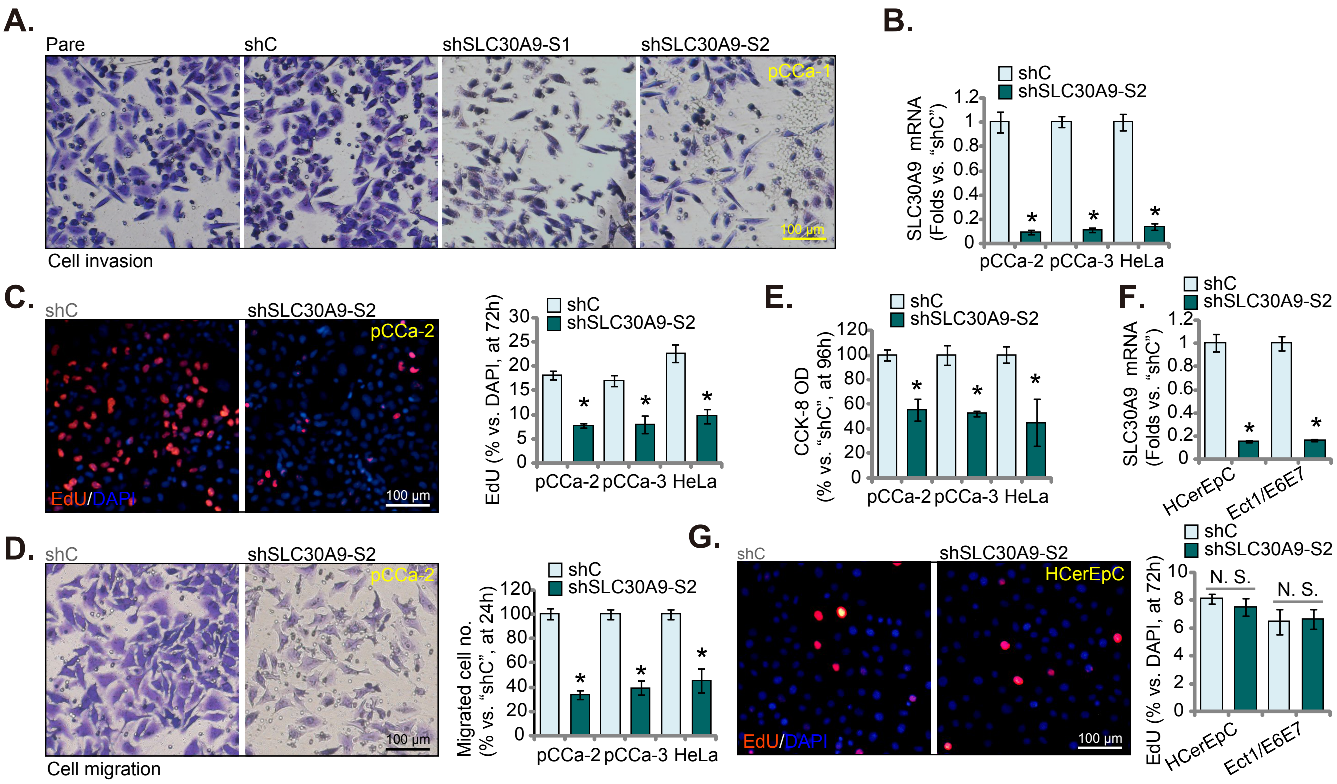
A.
Pare
shC
shSLC30A9-S1
shSLC30A9-S2
pCCa-1
100 μm
Cell invasion
B.
shC
shSLC30A9-S2
SLC30A9 mRNA (Folds vs. "shC")
pCCa-2 pCCa-3 HeLa
C.
shC
shSLC30A9-S2
pCCa-2
EdU/DAPI
100 μm
EdU (% vs. DAPI, at 72h)
D.
shC
shSLC30A9-S2
pCCa-2
100 μm
Cell migration
Migrated cell no. (% vs. "shC", at 24h)
E.
CCK-8 OD (% vs. "shC", at 96h)
F.
HCerEpC
Ect1/E6E7
G.
HCerEpC
N. S.
EdU (% vs. DAPI, at 72h)

Figure S4.

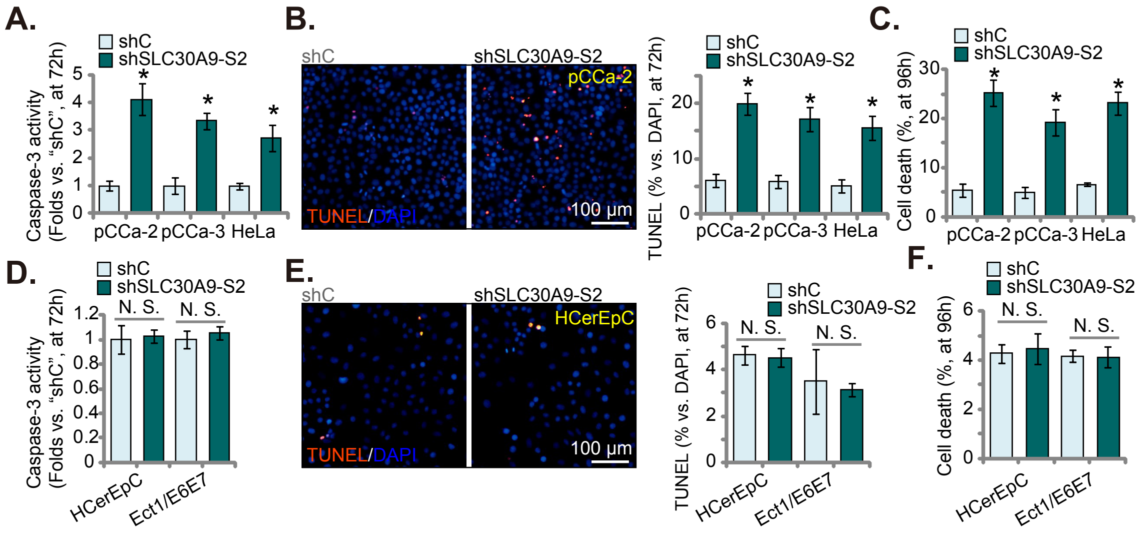
A.
shC
shSLC30A9-S2
Caspase-3 activity (Folds vs. "shC", at 72h)
pCCa-2 pCCa-3 HeLa
B.
shC
shSLC30A9-S2
pCCa-2
TUNEL/DAPI
100 μm
TUNEL (% vs. DAPI, at 72h)
C.
Cell death (%, at 96h)
D.
N. S.
HCerEpC
Ect1/E6E7
E.
HCerEpC
F.

Figure S5.

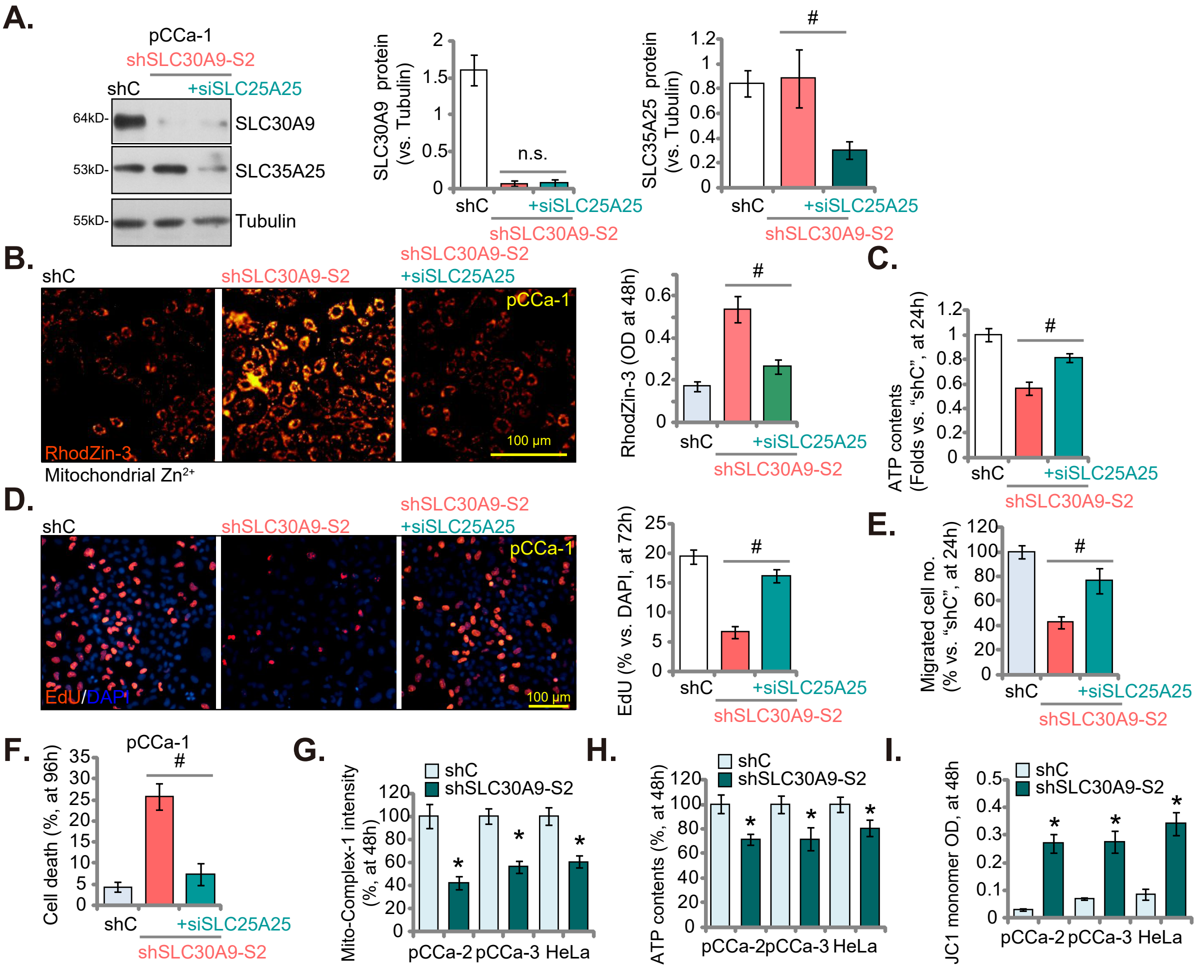

A.
pCCa-1
shSLC30A9-S2
shC
+siSLC25A25
64kD-
SLC30A9
53kD-
SLC35A25
55kD-
Tubulin
SLC30A9 protein (vs. Tubulin)
n.s.
SLC35A25 protein (vs. Tubulin)
#
B.
shC
shSLC30A9-S2
shSLC30A9-S2 +siSLC25A25
pCCa-1
RhodZin-3
Mitochondrial Zn2+
100 µm
RhodZin-3 (OD at 48h)
C.
ATP contents (Folds vs. "shC", at 24h)
D.
EdU/DAPI
EdU (% vs. DAPI, at 72h)
E.
Migrated cell no. (% vs. "shC", at 24h)
F.
Cell death (%, at 96h)
G.
Mito-Complex-1 intensity (%, at 48h)
pCCa-2
pCCa-3
HeLa
H.
ATP contents (%, at 48h)
I.
JC1 monomer OD, at 48h

Figure S6.

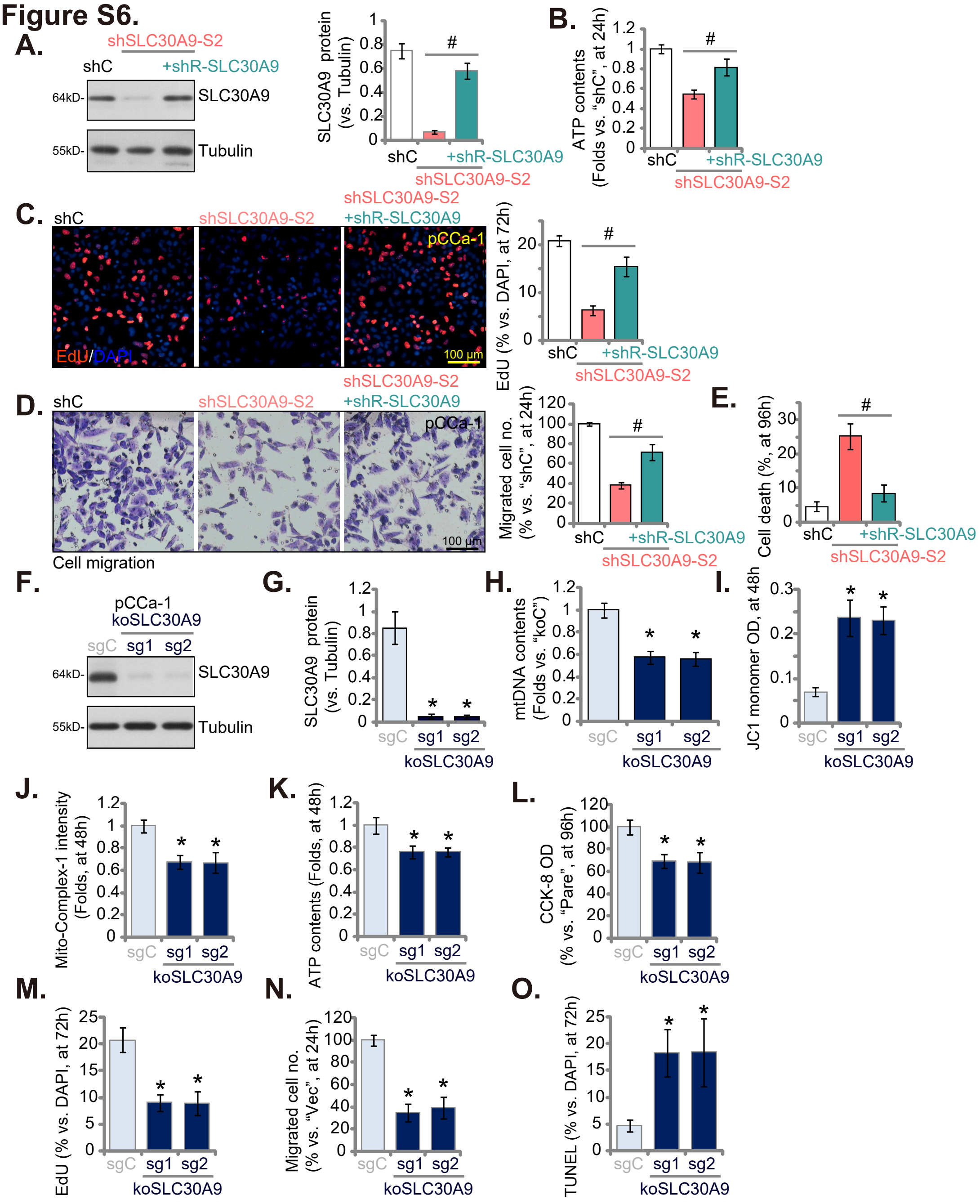

A.
shSLC30A9-S2
shC
+shR-SLC30A9
64kD-
SLC30A9
55kD-
Tubulin
SLC30A9 protein (vs. Tubulin)
#
B.
ATP contents (Folds vs. "shC", at 24h)
C.
pCCa-1
EdU/DAPI
100 µm
EdU (% vs. DAPI, at 72h)
D.
Cell migration
Migrated cell no. (% vs. "shC", at 24h)
E.
Cell death (%, at 96h)
F.
pCCa-1
koSLC30A9
sgC
sg1
sg2
G.
H.
mtDNA contents (Folds vs. "koC")
I.
JC1 monomer OD, at 48h
J.
Mito-Complex-1 intensity (Folds, at 48h)
K.
ATP contents (Folds, at 48h)
L.
CCK-8 OD (% vs. "Pare", at 96h)
M.
EdU (% vs. DAPI, at 72h)
N.
Migrated cell no. (% vs. "Vec", at 24h)
O.
TUNEL (% vs. DAPI, at 72h)

Figure S7

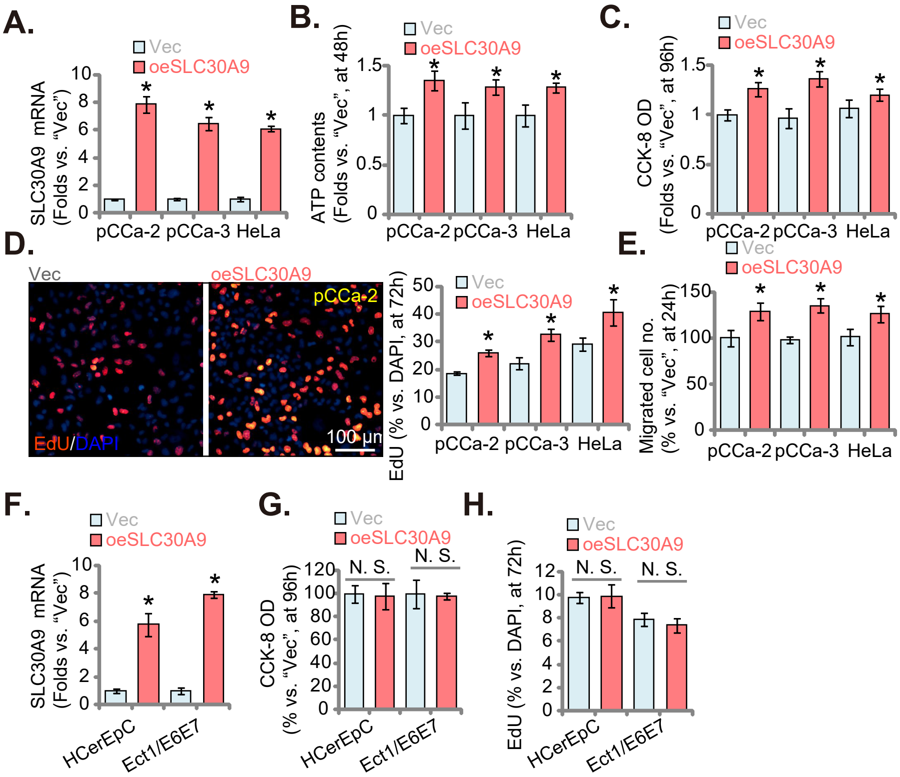
A.
Vec
oeSLC30A9
SLC30A9 mRNA
(Folds vs. "Vec")
pCCa-2 pCCa-3 HeLa
B.
ATP contents
(Folds vs. "Vec", at 48h)
C.
CCK-8 OD
(Folds vs. "Vec", at 96h)
D.
EdU/DAPI
pCCa-2
100 μm
EdU (% vs. DAPI, at 72h)
E.
Migrated cell no.
(% vs. "Vec", at 24h)
F.
HCerEpC
Ect1/E6E7
G.
N. S.
CCK-8 OD
(% vs. "Vec", at 96h)
H.
EdU (% vs. DAPI, at 72h)

Figure S8.

A.

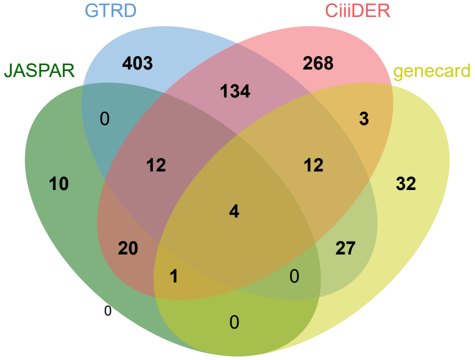

Four commonly predicted transcription factors:
PRDM1, PRDM4, SP1, and KLF9

B.

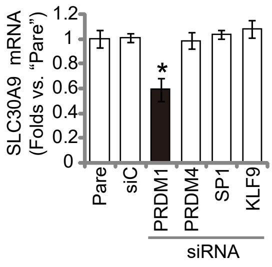

C.

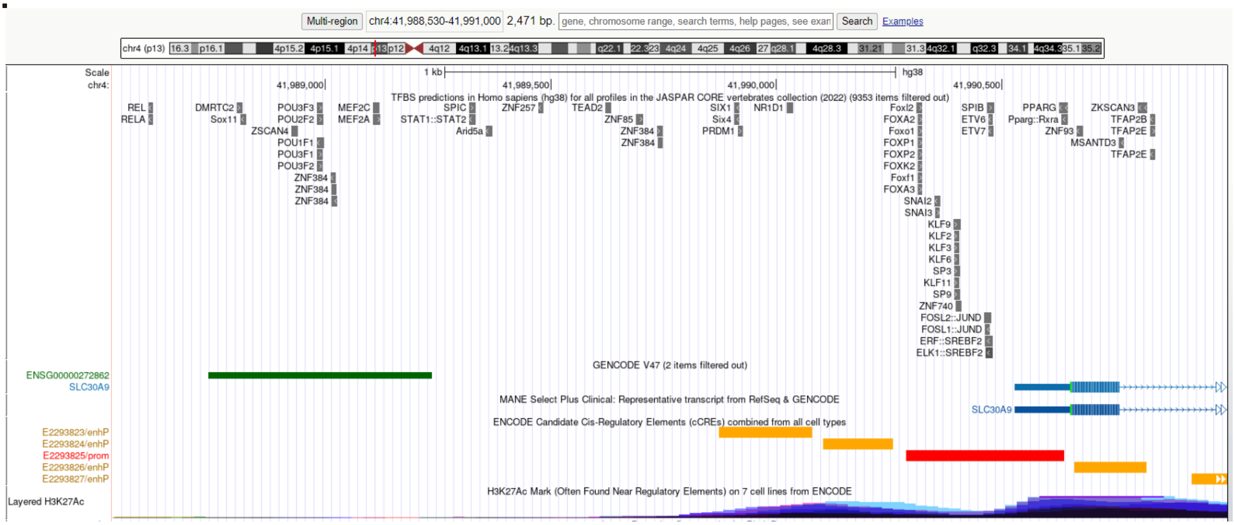

Color	UCSC label	ENCODE classification	ENCODE label
red	prom	promoter-like signature	PLS
orange	enhP	proximal enhancer-like signature	pELS
yellow	enhD	distal enhancer-like signature	dELS
pink	K4m3	DNase-H3K4me3	DNase-H3K4me3
blue	CTCF	CTCF-only	CTCF-only

Figure S1. The uncropped blotting images of the study.

Figure S2. UMAP plot showing 12 distinct cell clusters identified by graph-based clustering at a resolution of 0.1. Each cluster is color-coded and numbered (**A**). Dot plot showing the average expression and the percentage of cells expressing selected classical marker genes across the identified clusters (0-11) (**B**). The size of each dot corresponds to the percentage of cells in a cluster expressing the gene, and the color intensity represents the average scaled expression level. Feature plots illustrating the expression distribution of representative marker genes on the UMAP plot: *EPCAM* (Epithelial), *PECAM1* (Endothelial), *ACTA2* (Muscle), *COL1A2* (Fibroblast), *DCN* (Fibroblast), *LYZ* (Macrophage), *CD163* (Macrophage), *CD3D* (T cell), *CD2* (Lymphocyte), and *CSF3R* (Neutrophil). Color intensity indicates scaled expression levels, with red representing higher expression (**C**).

Figure S3. The patient-derived primary human cervical cancer cells (pCCa-1) were transduced with either the lentiviral SLC30A9 shRNA (“shSLC30A9-S1/shSLC30A9-S2,” representing two distinct shRNAs) or a scrambled control shRNA (“shC”), stable cells were formed after puromycin selection, cells were further cultured for 24h, and representative cell invasion images were shown (**A**); The patient-derived primary human cervical cancer cells (pCCa-2, and pCCa-3, from two other patients) (**B**-**E**), the established HeLa cell line (**B**-**E**), the primary human cervical epithelial cells (HCerEpC) (**F** and **G**) or Ect1/E6E7 cervical epithelial cell line (**F** and **G**) were transduced with shSLC30A9-S2 or shC, with stable cells formed after puromycin selection. The expression levels of specific mRNA were tested (**B** and **F**). Cells were further cultured in the basal medium for the applied time periods, cell proliferation (**C** and **G**), *in vitro* cell migration (**D**) and cell viability (CCK-8 OD, **E**) were measured. Time points at which these experiments were conducted were indicated on the Y-axis of the respective graphs. Results are expressed as mean ± standard deviation (SD, n=5, biological repeats). “Pare” stands for the parental control cells. Asterisks indicate significance with * P < 0.05 compared to the “shC” treatment. “N.S.” denotes no statistically significant difference (P > 0.05). The experiments were repeated five times with similar results obtained. Scale bar = 100 μm.

Figure S4. The patient-derived primary human cervical cancer cells (pCCa-2, and pCCa-3) (**A**-**C**), the established HeLa cell line (**A**-**C**), the primary human cervical epithelial cells (HCerEpC) (**D**-**F**) or Ect1/E6E7 cervical epithelial cell line (**D**-**F**) were transduced with

shSLC30A9-S2 or shC, with stable cells formed after puromycin selection. The Caspase-3 activity was measured (**A** and **D**). Cells were further cultured in the basal medium for the applied time periods, cell apoptosis and death were measured by the nuclear TUNEL staining (**B** and **E**) and the Trypan blue staining (**C** and **F**) assays, respectively. Time points at which these experiments were conducted were indicated on the Y-axis of the respective graphs. Results are expressed as mean ± standard deviation (SD, n=5, biological repeats). Asterisks indicate significance with * $P < 0.05$ compared to the "shC" treatment. "N.S." denotes no statistically significant difference ($P > 0.05$). The experiments were repeated five times with similar results obtained. Scale bar = 100 μm.

Figure S5. The patient-derived primary human cervical cancer cells (pCCa-1) with the lentiviral SLC30A9 shRNA ("shSLC30A9-S2") were further transduced with the specific siRNA targeting SLC25A25 ("siSLC25A25", 200 nM for two rounds, every 24h), control cells were with a scrambled control shRNA ("shC"). Expression of listed proteins were shown (**A**). Cells were further cultured in the basal medium for the applied time periods, several key parameters were evaluated, including mitochondrial Zn^{2+} levels (RhodZin-3 intensity, **B**), ATP contents (**C**), cell proliferation (EdU staining assays, **D**), cell migration ("Transwell assays", **E**) and overall cell death (via Trypan blue exclusion, **F**). The patient-derived primary human cervical cancer cells (pCCa-2, and pCCa-3) or the established HeLa cell line were transduced with shSLC30A9-S2 or shC, with stable cells formed after puromycin selection. Cells were further cultured in the basal medium for the applied time periods, mitochondrial complex I activity (**G**), ATP contents (**H**) and mitochondrial depolarization (**I**, via measuring JC-1 green monomers intensity) were tested. Time points at which these experiments were conducted were indicated on the Y-axis of the respective graphs. Results are expressed as mean ± standard deviation (SD, n=5, biological repeats). $^{\#}P < 0.05$ vs. "shSLC30A9-S2" only treatment (**A**-**F**). * $P < 0.05$ compared to the "shC" treatment (**G**-**I**). The experiments were repeated five times with similar results obtained. Scale bar = 100 μm.

Figure S6. The patient-derived primary human cervical cancer cells (pCCa-1) with the lentiviral SLC30A9 shRNA ("shSLC30A9-S2") or together with an shRNA-resistant SLC30A9 cDNA-expressing construct (+shR-SLC30A9), control cells were with a scrambled control shRNA ("shC"). Expression of listed proteins were shown (**A**). Cells were further cultured in the basal medium for the applied time periods, several key parameters were evaluated, including ATP contents (**B**), cell proliferation (EdU assays, **C**), cell migration ("Transwell

assays”, **D**) and overall cell death (via Trypan blue exclusion, **E**). The Cas9-expressing pCCa-1 primary cervical cancer cells were transduced with the lenti-CRISPR/Cas9 construct encoding sgRNA sequences against human SLC30A9: koSLC30A9-sg1 and koSLC30A9-sg2, or the lenti-CRISPR/Cas9 construct with non-sense sgRNA (“sgC”). Stable cells were formed. The protein expression level of SLC30A9 was then assessed (**F** and **G**). Cells were further cultured in the basal medium for the applied time periods, the mtDNA contents were measured (**H**). Mitochondrial depolarization was determined by JC-1 green monomer intensity (**I**); The mitochondrial complex I activity (**J**) and cellular ATP contents (**K**) were also tested. Additionally, cell viability (CCK-8 OD, **L**), cell proliferation (by measuring EdU-positive nuclei ratio, **M**), migration (“Transwell” assays, **N**) and apoptosis (TUNEL-positive nuclei ratio, **O**) were also measured. Time points at which these experiments were conducted were indicated on the Y-axis of the respective graphs. Results are expressed as mean ± standard deviation (SD, n=5, biological repeats). $^{\#}$***P*** < 0.05 vs. “shSLC30A9-S2” only treatment (**A**-**E**). * ***P*** < 0.05 compared to the “sgC” treatment (**F**-**O**). The experiments were repeated five times with similar results obtained. Scale bar = 100 μm.

Figure S7. The patient-derived primary human cervical cancer cells (pCCa-2, and pCCa-3) (**A**-**E**), the established HeLa cell line (**A**-**E**), the primary human cervical epithelial cells (HCerEpC) (**F**-**H**) or Ect1/E6E7 cervical epithelial cell line (**F**-**H**) were transduced with the same lentiviral construct expressing SLC30A9 (“oeSLC30A9”) or the empty vector (“Vec”), with stable cells formed after puromycin selection. Expression of *SLC30A9* mRNA was shown (**A** and **F**); Cells were further cultured in the basal medium for the applied time periods, ATP contents (**B**), cell viability (**C** and **G**), proliferation (**D** and **H**) and *in vitro* cell migration (**E**) were tested similarly. Time points at which these experiments were conducted were indicated on the Y-axis of the respective graphs. Results are expressed as mean ± standard deviation (SD, n=5, biological repeats). Asterisks indicate significance with * ***P*** < 0.05 compared to the “shC” treatment. “N.S.” denotes no statistically significant difference (***P*** > 0.05). The experiments were repeated five times with similar results obtained. Scale bar = 100 μm.

Figure S8. Venn diagram illustrating the number of potential transcription factors predicted to bind to the regulatory region of the *SLC30A9* gene by four independent databases: UCSC-JASPAR (green), GTRD (blue), GeneCard (yellow), and CiiiDER (red). The overlapping regions indicate the number of transcription factors predicted by multiple databases (**A**). The central intersection shows the four transcription factors commonly predicted by all four

databases (**A**). pCCa-1 cells were transfected with verified siRNA at 200 nM through two sequential rounds, each lasting 24h. Subsequently, the expression levels of the listed mRNAs were determined (**B**). UCSC Genome Browser view of the *SLC30A9* gene region (chr4:41988530-41991000, hg38 reference genome), encompassing the defined promoter area. Tracks displayed include: Gene structure of *SLC30A9*, predicted transcription factor binding sites from the JASPAR database, ENCODE candidate cis-regulatory elements (CREs) indicating potential promoter-like (red), proximal enhancer-like (orange), and distal enhancer-like (yellow) regions, as well as other regulatory classifications, and layered H3K27ac data reflecting active enhancer and promoter sites (**C**). Results are expressed as mean ± standard deviation (SD, n=5, biological repeats). * $\boldsymbol{P} < 0.05$ compared to the scramble siRNA "siC" treatment. The experiments were repeated five times with similar results obtained. Scale bar = 100 μm.
